# Processive kinetics in the three-step lanosterol 14α-demethylation reaction catalyzed by human cytochrome P450 51A1

**DOI:** 10.1016/j.jbc.2023.104841

**Published:** 2023-05-18

**Authors:** Kevin D. McCarty, Molly E. Sullivan, Yasuhiro Tateishi, Tatiana Y. Hargrove, Galina I. Lepesheva, F. Peter Guengerich

**Affiliations:** Department of Biochemistry, Vanderbilt University School of Medicine, Nashville, Tennessee, USA

**Keywords:** cytochrome P450, lanosterol demethylase, steroid biosynthesisP450 51A1, CYP51, lanosterol, sterol demethylation, steroidogenesis, enzyme kinetics, pre-steady-state kinetics, reaction processivity, multi-step reaction

## Abstract

Cytochrome P450 (P450, CYP) family 51 enzymes catalyze the 14α-demethylation of sterols, leading to critical products used for membranes and the production of steroids, as well as signaling molecules. In mammals, P450 51 catalyzes the 3-step, 6-electron oxidation of lanosterol to form (4β,5α)-4,4-dimethyl-cholestra-8,14,24-trien-3-ol (FF-MAS). P450 51A1 can also use 24,25-dihydrolanosterol (a natural substrate in the Kandutsch-Russell cholesterol pathway). 24,25-Dihydrolanosterol and the corresponding P450 51A1 reaction intermediates, the 14α-alcohol and -aldehyde derivatives of dihydrolanosterol, were synthesized to study the kinetic processivity of the overall 14α-demethylation reaction of human P450 51A1. A combination of steady-state kinetic parameters, steady-state binding constants, dissociation rates of P450-sterol complexes, and kinetic modeling of the time course of oxidation of a P450-dihydrolanosterol complex showed that the overall reaction is highly processive, with *k*_off_ rates of P450 51A1-dihydrolanosterol and the 14α-alcohol and 14α-aldehyde complexes being 1 to 2 orders of magnitude less than the forward rates of competing oxidations. epi-Dihydrolanosterol (the 3α-hydroxy analog) was as efficient as the common 3β-hydroxy isomer in the binding and formation of dihydro FF-MAS. The common lanosterol contaminant dihydroagnosterol was found to be a substrate of human P450 51A1, with roughly one-half the activity of dihydrolanosterol. Steady-state experiments with 14α-methyl deuterated dihydrolanosterol showed no kinetic isotope effect, indicating that C-14α C-H bond breaking is not rate-limiting in any of the individual steps. The high processivity of this reaction generates higher efficiency and also renders the reaction less sensitive to inhibitors.

Many enzymes catalyze multiplestep reactions, that is, an initial product is the substrate for a subsequent reaction. This phenomenon is not surprising, in that many products are highly similar to the parent that leads to them. Such “intermediate” products may either remain bound in the active site throughout the overall reaction or may be released after completion of each step. Notable examples are DNA and RNA polymerases and various hydrolytic enzymes such as nonspecific proteases and enzymes that degrade starches. A general consideration for multistep enzymes is their degree of processivity, that is, the proclivity to remain bound to a substrate/product as opposed to being released from the enzyme and rebinding. This is not simply a pedantic issue but also a practical one. In terms of enzyme engineering, a case can be made that a highly processive enzyme should be more efficient. Moreover, an enzyme should be harder to inhibit if it remains bound to a substrate, in that the active site is precluded. The extreme cases are a highly processive enzyme—which remains tightly bound to its substrates through many cycles (*e.g.*, DNA polymerase)—and a distributive enzyme, which releases a product that it must rebind to perform the next step.

Cytochrome P450 (P450, CYP) enzymes are involved in more known oxidation reactions than any other enzymes (1). They are the principal catalysts involved in the metabolism of steroids, drugs, fat-soluble vitamins, and natural products (2). Multistep P450 reactions are common in natural product biosynthesis (3) and also in the metabolism of drugs (4, 5). For instance, it is not unusual for a single drug to be degraded to ≥10 metabolites, with individual P450s catalyzing multiple steps (4, 5, 6, 7). Multistep P450 oxidations are also common in the metabolism of steroids and fat-soluble vitamins, for example, P450s 11A1, 11B1, 11B2, 17A1, 19A1, 24A1, 26A1, and 51A1 (8). Previous work in this laboratory has shown that, at least with the substrates examined, P450 2E1 is processive (9, 10), P450 19A1 is distributive (11), P450 17A1 has a mixed processive and distributive nature (12), and P450 11B2 is processive (and has an unusual pattern in that one of the products effectively locks into a stable acetal (lactol) form) (13).

P450 enzymes in Family 51 (all of which are sterol 14α-demethylases) are important in that they catalyze a necessary C-C bond-breaking demethylation in the biosynthesis of critical sterols (14, 15). In mammals, this step results in the 14α-demethylation of lanosterol and 24,25-dihydrolanosterol (dihydrolanosterol), enroute to the essential molecule cholesterol (16). The P450 51 enzymes in fungi, yeasts, and protozoan parasites are important drug targets, and most azoles act by inhibiting these enzymes and thus affecting the production of ergosterol and ergosterol-like molecules (17). The overall reaction sequence catalyzed by P450 51 in mammals, with dihydrolanosterol as the first substrate, is shown in Figure 1. In cells, the reduction of the 24,25-double bonds of sterols is catalyzed by the enzyme dehydrocholesterol reductase (DHCR24).

**Figure 1:**
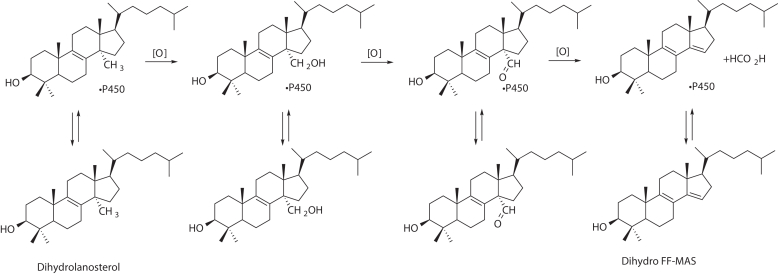
**Three-step oxidation of dihydrolanosterol to dihydro FF-MAS.** The equilibria between free and enzyme-bound sterols are shown, and the rates are the subject of this article. FF-MAS, follicular fluid meiosis-activating sterol ((4β,5α)-4,4-dimethyl-cholesta-8,14,24-trien-3-ol).

Although numerous X-ray crystal structures of human and other P450 51 enzymes are available, there are still a number of relevant questions about details of the catalytic mechanism. We have addressed some of these, including the processivity of the reaction. Very little has been reported in this regard, aside from a study with rat liver microsomes and excess dihydrolanosterol (50 μM), in which low levels of the 14α-CH_2_OH and 14α-CHO products accumulated in 2 h incubations (18). We had previously reported that the 14α-CHO derivative of lanosterol could be converted to the final product, (4β,5α)-4,4-dimethyl-cholestra-8,14,24-trien-3-ol (follicular fluid meiosis-activating factor, [FF-MAS]), by human P450 51A1 (19).

In this work, we synthesized a number of critical compounds and addressed the roles of epi-dihydrolanosterol and the natural contaminant dihydroagnosterol (Fig. 2), the issue of whether a kinetic deuterium isotope effect occurs, and how processive the overall reaction is. Pre-steady-state (rapid quench) kinetic methods were used to follow the course of a single turnover of human P450 51A1, and computational simulations were used to develop an acceptable model that also fit the bounds of steady-state kinetic analyses and determinations of binding constants. The results reveal that the overall reaction is highly processive.

**Figure 2:**
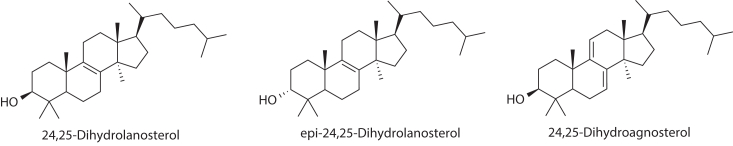
**Structures of dihydrolanosterol (Δ**^**8**^**), epi-dihydrolanosterol, and dihydroagnosterol**.

## Results

### Development of HPLC-UV assays

Most assays of P450 51A1 catalytic activity have utilized radio-HPLC (or TLC) approaches (20). We designed a method that did not require radioisotopes and could be utilized with intermediate products (*i.e.*, the 14α-CH_2_OH and 14α-CHO products of dihydrolanosterol) as substrates leading to dihydro FF-MAS.

**Figure 3:**
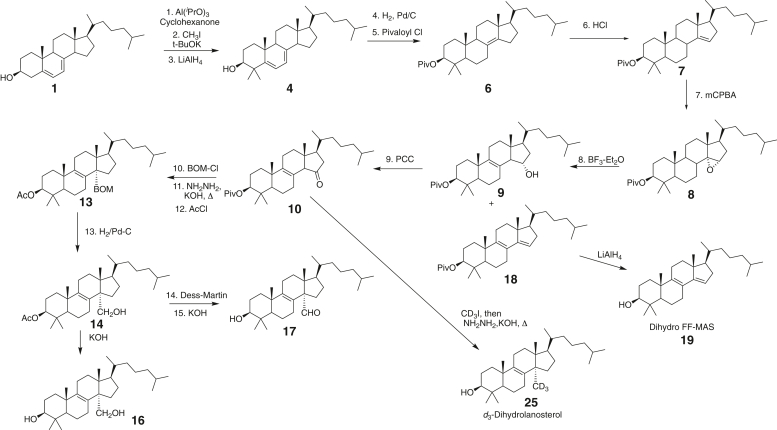
**Abbreviated synthetic routes.**Fig. S1. The numbering used for individual components in Fig. S1 is also used here.

Our approach to the synthesis of oxygenated P450 51 reaction intermediates (Figs. 3 and S1) involved the use of 24,25-dihydrolanosterol instead of lanosterol (21, 22, 23). These two compounds are involved in the Kandutsch–Russell and Bloch pathways of cholesterol biosynthesis, respectively (15, 24), and the rates of 14α-demethylation by human P450 51A1 are similar (20). The presence of the 24,25-olefin is problematic in the synthesis of modified lanosterol derivatives (due to lability to oxidation), and commercial sources of natural lanosterol are generally mixtures of lanosterol and dihydrolanosterol (25).

We worked exclusively with dihydrolanosterol as the starting substrate in this study (Fig. 1). Dihydrolanosterol is readily prepared from lanosterol by catalytic hydrogenation (22, 26).

We first attempted the synthesis of the oxygenated sterols of interest (14α-alcohol and 14α-aldehyde) using a combination of the approaches of Parish and Schroepfer (26) and Sonoda *et al.* (27) but found the separation of Δ^8^- and Δ^7^-sterols difficult. Accordingly, we switched to a scheme outlined by Morisaki *et al.* (21, 22, 23) that began with commercially available 7-dehydrocholesterol. The 16-step synthesis of various oxygenated and other derivatives of dihydrolanosterol (Figs. 3 and S1) was long, but the individual steps are relatively straightforward and most products were obtained in good yield. The key intermediates 12 and 14 (Figs. 3 and S1–S16; Table S1) were characterized in detail with high-resolution mass spectrometry and NMR, and the NOESY spectra established that the addition at C-14 was in the α configuration, as proposed earlier (21).

The final enzymatic product FF-MAS (Fig. 1) has been synthesized previously (28, 29), but in our synthesis of 14α-oxygenated dihydrolanosterol derivatives (Figs. 3 and S1), we found that a side product with strong UV absorbance was formed in the step involving BF_3_ treatment of the 14,15-epoxide compound **8** (Figs. 3 and S17). This compound was identified as the 3-pivaloyl ester of dihydro FF-MAS (compound **19**). The pivaloyl ester resisted hydrolysis under the usual hydrolysis methods (KOH-CH_3_OH-benzene-heat) but could be readily cleaved using LiAlH_4_ to yield dihydro FF-MAS.

HPLC showed the presence of two adjacent peaks with identical UV and mass spectra. The enzymatic product (generated from dihydrolanosterol by P450 51A1) eluted with the slower migrating of these. We characterized the faster eluting peak as the 3α-hydroxy form of dihydro FF-MAS by its different chemical shift in ^1^H-NMR (δ 3.30 *versus* δ 3.25). Also, when epi-lanosterol was incubated with P450 51, what we assigned to be epi-FF-MAS has a t_R_ identical to that of this peak. We propose that this epi-form of dihydro FF-MAS might have been generated in the course of the LiA1H_4_ ester cleavage due to trace moisture and the activation of hydroxide ions by Li^+^ (Fig. S19).

**Figure 4:**
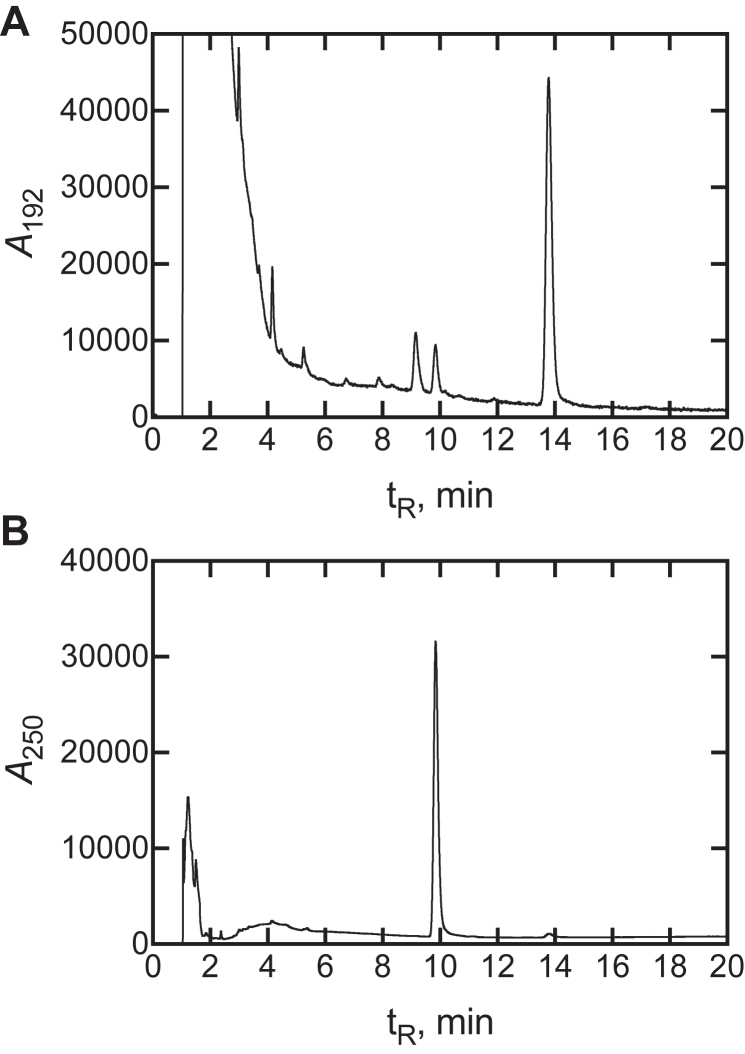
**LC-UV assay of conversion of dihydrolanosterol oxidation to dihydro FF-MAS.** UPLC traces of a reaction of 10 μM dihydrolanosterol with 0.05 μM P450 51A1 for 5 min. The products were extracted and analyzed by UPLC. *A*, *A*_192_; *B*, *A*_250_ measurements. Peaks for the residual lanosterol after the reaction (*A*, t_R_ 14.1 min) and the demethylated product, dihydro FF-MAS (*B*, t_R_ 9.9 min), are shown. The intermediates (14α-alcohol and 14α-aldehyde) were not detected in steady-state experiments but absorb maximally at 192 nm and 230 nm, respectively. The *A*_250_ character of the dihydro FF-MAS product is the result of the additional degree of unsaturation of the diene. FF-MAS, follicular fluid meiosis-activating sterol ((4β,5α)-4,4-dimethyl-cholesta-8,14,24-trien-3-ol); UPLC, ultra-performance liquid chromatography.

FF-MAS has a relatively high extinction coefficient (ε_249_ 18,600 M^−1^ cm^−1^) (28), as high as for Δ4 steroids (30), and its separation from other P450 51A1 sterol products using HPLC and the relatively rapid rate of formation afforded a convenient and inexpensive method of monitoring reactions (Fig. 4), based on Sonoda *et al.* (31), which was used extensively in these studies.

### Lack of kinetic hydrogen isotope effect

The synthetic route used to prepare 14α-oxygenated products afforded an opportunity to prepare 14α-CD_3_ (32-*d*_3_) dihydrolanosterol (Figs. 3 and S21). The final product was further purified using a semipreparative HPLC column to remove contaminating 14-desmethyl dihydrolanosterol (generated due to lack of complete reaction of CD_3_I with compound 10 in Fig. S21).

Repeated analyses yielded no substantial difference in the rate of conversion of *d*_0_ and *d*_3_ dihydrolanosterol to dihydro FF-MAS at high substrate concentrations (10–15 μM). More detailed analysis yielded *k*_cat_/*K*_m_ values of 1.1 (±0.03) × 10^5^ M^−1^ s^−1^ for *d*_0_-dihydrolanosterol and 1.3 (±0.2) × 10^5^ M^−1^ s^−1^ for *d*_3_-dihydrolanosterol (data not presented). We conclude that C-H bond breaking at C-14 is not a rate-limiting step in any of the individual P450 51A1 reactions (Fig. 1).

### epi-Dihydrolanosterol and dihydroagnosterol are also substrates of human P450 51A1

**Figure 5:**
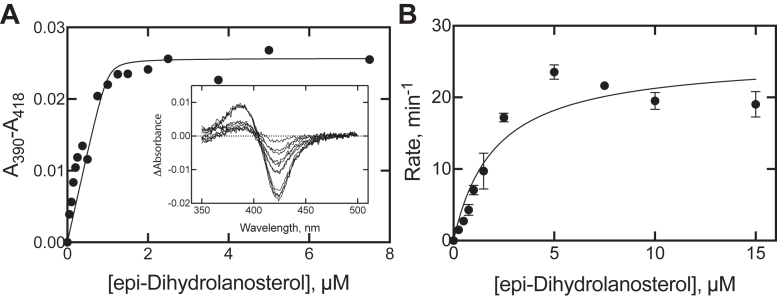
**Binding of epi-dihydrolanosterol and steady-state kinetics of oxidation of epi-dihydrolanosterol to epi-dihydro FF-MAS.***A*, *K*_d_ 0.014 (±0.031) μM. *B*, *k*_cat_ 0.42 ± 0.03 s^−1^, *K*_m_ 2.0 ± 0.4 μM. Rates are presented on the y-axis as nmol product (epi-dihydro FF-MAS) formed min^−1^ (nmol P450)^−1^ in (*B*). Incubations were run in triplicate, and the means (±SD) were calculated and plotted. The linear regression fits include the Prism error estimates for internal fitting (SE). FF-MAS, follicular fluid meiosis-activating sterol ((4β,5α)-4,4-dimethyl-cholesta-8,14,24-trien-3-ol).

In the course of synthesis of dihydrolanosterol derivatives, we observed that the reduction of the 3-keto compound (derived from oxidation of dihydrolanosterol) by NaBH_4_ yielded the α- and β-hydroxy products in a 1:9 ratio (Fig. S20), consonant with other synthetic work on sterols with LiAlH_4_ (32) and NaBH_4_ (33). The α-hydroxy product is termed epi-lanosterol (32). It was shown to be a substrate for yeast P450 51 (34), but its activity with the human enzyme had not been explored.

epi-Dihydrolanosterol produced a low- to high-spin shift in the P450 iron and gave a classic type I spectral shift seen often with substrates (Fig. 5) (35). It bound tightly to human P450 51A1 and was also oxidized to (epi)-dihydro FF-MAS slightly more efficiently (than dihydrolanosterol) (Fig. 5 ). In the crystal structure of human P450 51A1 that we previously determined in complex with lanosterol (Protein Data Bank code 6UEZ), the C3β-OH of the sterol molecule interacts with the main chain oxygen of Ile-379, and modeling of epi-dihydrolanosterol in this structure suggests that the H-bond between the enzyme and C3α-OH could be stronger (data not shown).

Agnosterol (Fig. 2) is a common Δ^7,14^ contaminant of commercial lanosterol preparations (Fig. S22) (25, 36). We also encountered this molecule and its derivatives, with its characteristic UV spectra derived from our synthesis beginning with 7-dehydrocholesterol (Figs. S1 and S17). The most likely entry point is the *m*-chloroperbenzoic acid epoxidation (Fig. S1, step 7) (37). The level of contamination was ∼2% in synthetic derivatives of dihydrolanosterol, as judged by either direct UV spectral analysis or LC radiometric analysis (of [3-^3^H]-dihydrolanosterol). The contaminant could be removed by preparative HPLC (C_18_, CH_3_CN, see Supporting Information). Comparisons indicated that this level of contamination did not influence rates of oxidation of dihydrolanosterol to dihydro FF-MAS (the estimated IC_50_ value was >10 μM under these conditions).

**Figure 6:**
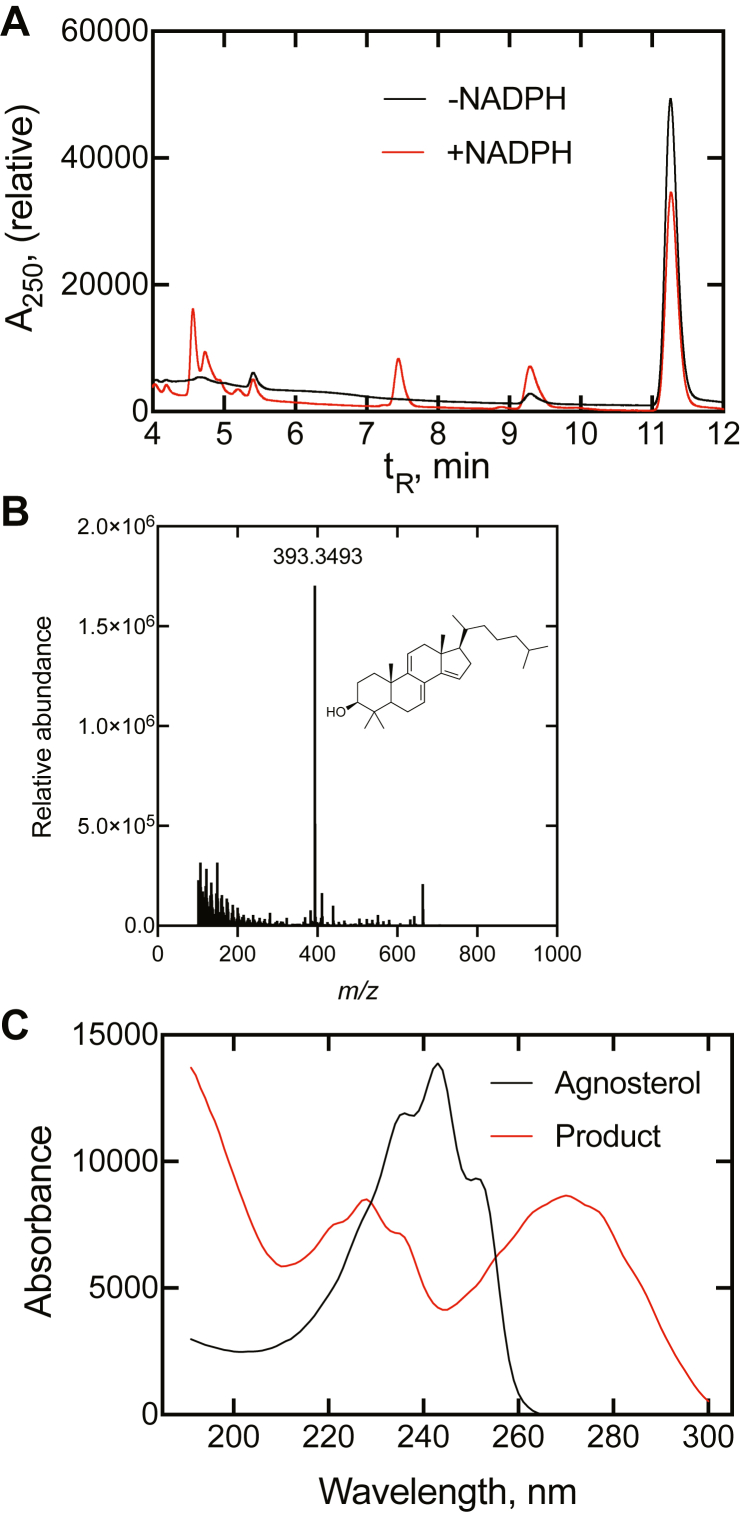
**Oxidation of dihydroagnosterol by human P450 51A1.** The incubation included 0.1 μM P450 51A1 and 10 μM dihydroagnosterol and ran for 10 min at 37 °C in the absence and presence of NADPH. The products were extracted and analyzed by UPLC-UV and UPLC-MS. *A*, UPLC chromatogram (UV, 192 and 245 nm). *B*, mass spectrum [M+H-H_2_O]^+^, calculated for C_29_H_45_, 393.3516; found, 393.3493 (−5.9 ppm) and assigned structure. *C*, UV spectrum (from t_R_ 7.3 min peak of A). UPLC, ultra-performance liquid chromatography.

The possibility existed that this molecule could be a substrate. Incubation of (isolated) dihydroagnosterol with human P450 51A1 resulted in two products as judged by LC-UV (rate of disappearance ∼10 min^−1^, ∼ one-half that of dihydrolanosterol) (Fig. 6). The amount of dihydroagnosterol was very limited, and we did not do an incubation on a scale sufficient to completely characterize the major product. However, on the basis of the UV and mass spectra and literature precedents (38, 39), we have assigned the structure as 4,4′-dimethylcholesta-7,9,14-trienol. The UV spectrum retains (Fig. 6) the detailed chromophore of agnosterol (Fig. S23) plus an additional transition at higher wavelength, as expected based on the UV spectrum of FF-MAS (Fig. S18).

### Steady-state measurements of P450 51A1 activity

**Figure 7:**
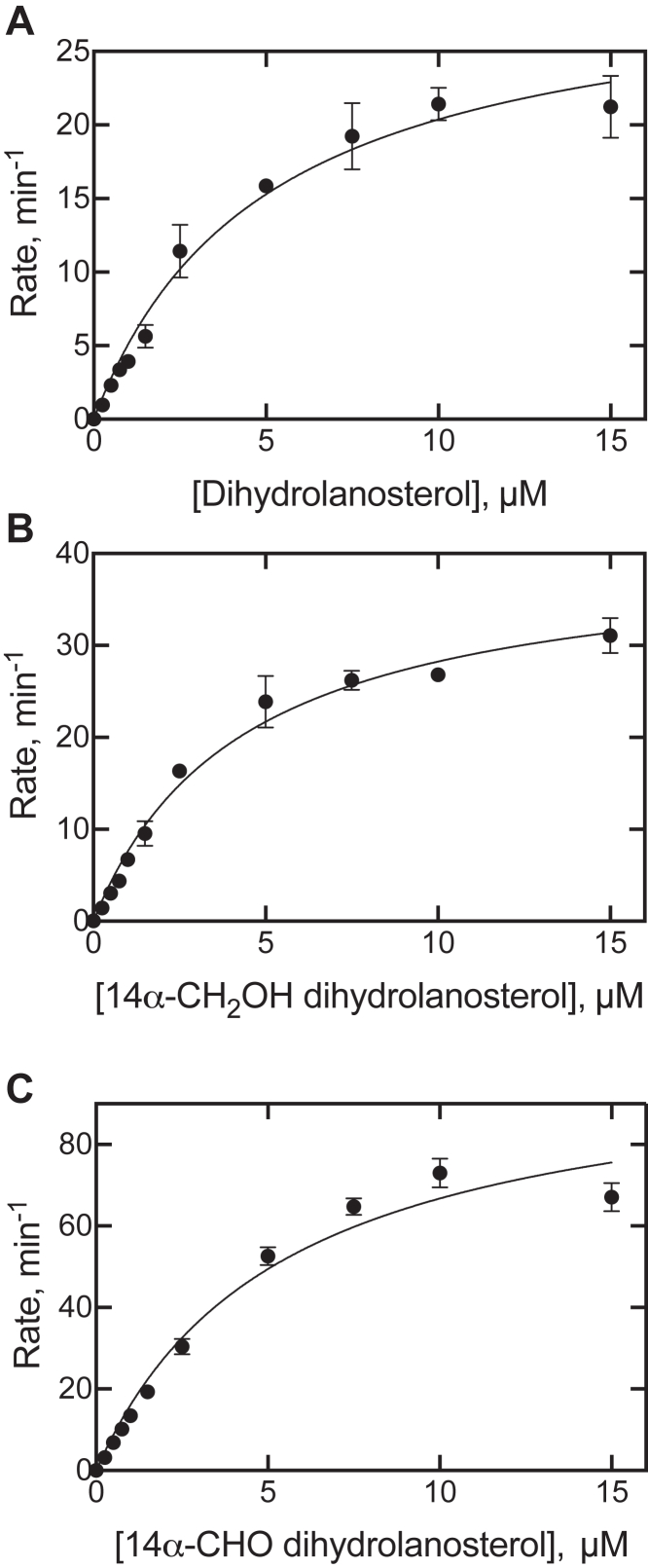
**Steady-state kinetics of oxidation of dihydrolanosterol and its 14α-CH**_**2**_**OH and 14α-CHO derivatives to dihydro FF-MAS.** The individual substrates were incubated with a reconstituted P450 51A1 system, and the product dihydro FF-MAS was extracted and analyzed by UPLC-UV. *A*, dihydrolanosterol: *k*_cat_ 0.50 ± 0.03 s^−1^, *K*_m_ 5.0 ± 0.6 μM; *B*, 14α-CH_2_OH dihydrolanosterol: *k*_cat_ 0.67 ± 0.03 s^−1^, *K*_m_ 4.3 ± 0.4 μM; *C*, 14α-CHO dihydrolanosterol: *k*_cat_ 1.7 ± 0.1 s^−1^, *K*_m_ 5.4 ± 0.7 μM. Rates are presented on the y-axis as nmol product (dihydro FF-MAS) formed min^−1^(nmol P450)^−1^. Incubations were run in triplicate, and the means (±SD) were calculated and plotted, The linear regression fits include the Prism error estimates for internal fitting (SE). The estimated parameters were not corrected using a quadratic equation, due to the high *K*_m_ values. FF-MAS, follicular fluid meiosis-activating sterol ((4β,5α)-4,4-dimethyl-cholesta-8,14,24-trien-3-ol); UPLC, ultra-performance liquid chromatography.

The LC-UV assay provided a convenient means of monitoring the conversion of dihydrolanosterol and its oxidation products (14α-CH_2_OH, 14α-CHO) to dihydro FF-MAS (Fig. 7). Steady-state kinetic analysis showed the oxidation of dihydrolanosterol and its 14α-CH_2_OH product to be similar and to be hyperbolic, with the latter being ∼1.5-fold higher for both *k*_cat_ and *k*_cat_/*K*_m_ (Fig. 7). The *k*_cat_ and *k*_cat_/*K*_m_ values beginning the reaction with the aldehyde were, in turn, about 3-fold higher than for dihydrolanosterol.

### Binding of sterols to P450 51A1

**Figure 8:**
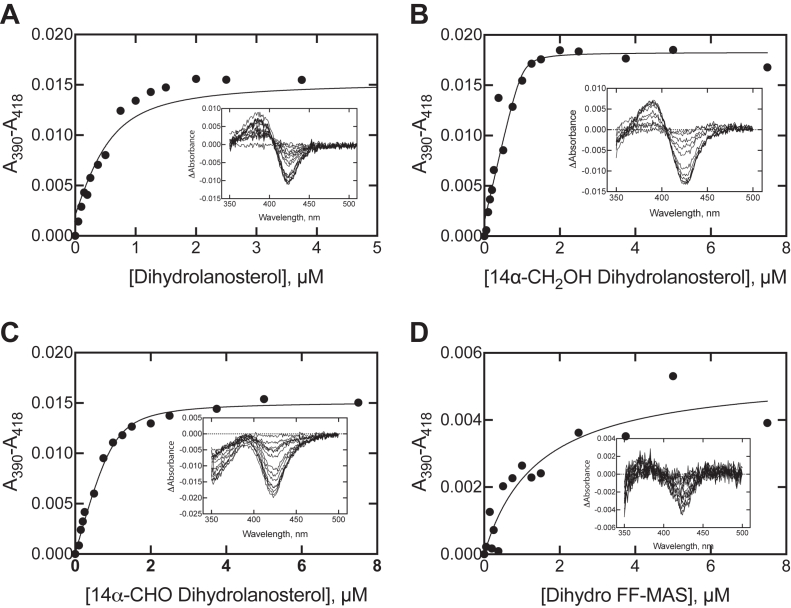
**Spectrally determined *K***_**d**_**values of P450 51A1–sterol complexes.** Two cuvettes containing 1.0 μM P450 51A1 were placed in a spectrophotometer, and the indicated concentrations of the individual compounds were added to the sample cuvette. The insets show spectral traces used in each analysis. The data were fit to a hyperbolic curve, using a quadratic equation due to the low *K*_d_ values. *A*, dihydrolanosterol (*K*_d_ 0.23 ± 0.10 μM); *B*, 14α-CH_2_OH dihydrolanosterol (*K*_d_ 0.02 ± 0.04 μM); *C*, 14α-CHO dihydrolanosterol (*K*_d_ 0.12 ± 0.03 μM); *D*, dihydro FF-MAS (*K*_d_ 1.4 ± 0.5 μM). FF-MAS, follicular fluid meiosis-activating sterol ((4β,5α)-4,4-dimethyl-cholesta-8,14,24-trien-3-ol).

All of the sterols in the pathway (Fig. 1) produced a low- to high-spin iron shift in the P450 51A1 spectrum, and the changes could be used to estimate *K*_d_ values (Fig. 8). The estimated values for dihydrolanosterol and the 14α-CH_2_OH and 14α-CHO derivatives were sub-μM. The plot for dihydro FF-MAS yielded a *K*_d_ of 1.4 μM (±0.5 μM), with the amplitude of the spectral changes being less than with the other sterols. The other *K*_d_ values were much lower than the enzyme concentration used (1 μM) and, even after application of quadratic fitting, contain considerable uncertainty. Subsequently, *k*_off_ rates were measured (see below) and are consonant with the low *K*_d_ values and more definitive.

### Sterol k_off_ measurements

Ketoconazole is known to bind tightly to human P450 51A1 (40, 41, 42), producing a type II spectral change (due to binding of an azole nitrogen to the heme iron), in an opposite direction to that generated by sterol binding (Fig. 8). Mixing of 10 μM ketoconazole with (1 μM) P450 51A1 (final concentrations) resulted in a spectral change with a pseudo-first-order rate of 25 s^−1^ (data not shown). Accordingly, the tight binding of the sterols to P450 51A1 allowed for experiments in which P450–sterol complexes could be mixed with 10 μM ketoconazole, which served as a trap for the free P450 and yielded *k*_off_ rates (43).

**Figure 9:**
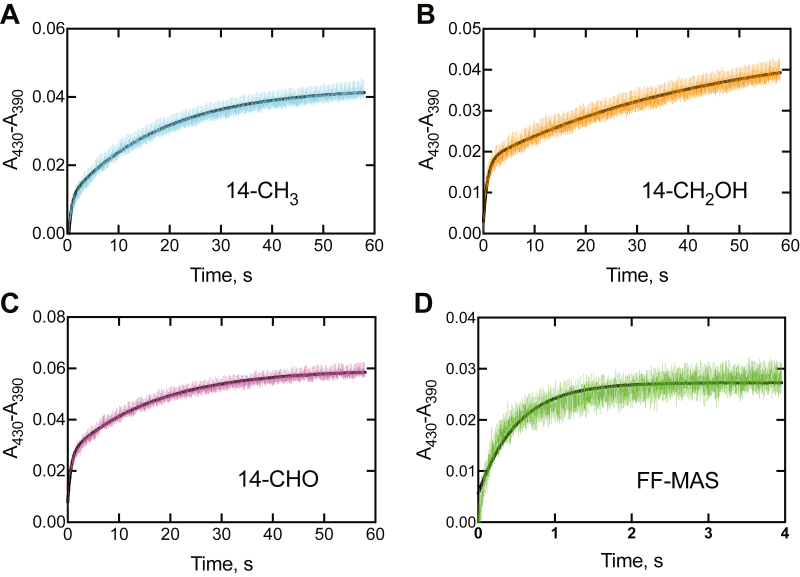
***k***_**off**_**measurements of P450 51A1–sterol complexes.** In each case, an equimolar concentration of P450 51A1 and each sterol, in one syringe, were mixed with a 20 μM concentration of ketoconazole in the other syringe, in an OLIS RSM1000 stopped-flow spectrophotometer. Full spectra were collected, and the *A*_390_ and *A*_430_ data were used in the calculations. At least five individual traces were averaged. Fits were to single exponentials using the OLIS GlobalWorks program. The error estimates were made (for each curve) in the program. *A*, dihydrolanosterol, 0.054 ± 0.010 s^−1^; *B*, 14α-CH_2_OH dihydrolanosterol, 0.022 ± 0.001 s^−1^; *C*, 14α-CHO dihydrolanosterol, 0.059 ± 0.001 s^−1^; *D*, dihydro FF-MAS, 2.0 ± 0.1 s^−1^. In (*D*), the change observed after 60 s was not much greater than shown at 4 s (≥90% complete), and accordingly, the 4 s trace was used in the calculation of the *k*_off_ rate. FF-MAS, follicular fluid meiosis-activating sterol ((4β,5α)-4,4-dimethyl-cholesta-8,14,24-trien-3-ol).

*k*_off_ rates were measured for the sterols in the reaction pathway (Fig. 1). In each case, an initial fast phase corresponding to ∼20% of the total amplitude was observed, attributed to the fraction of unliganded P450 51A1, followed by a slower terminal dissociation of the substrate/product from the enzyme prior to fast trapping as the ketoconazole complex. All *k*_off_ rates were << *k*_cat_ for the respective oxidations to dihydro FF-MAS (Fig. 9, *A*–*C*), with the exception of the final product dihydro FF-MAS (Fig. 9*D*) (*k*_off_ 2.0 s^−1^). In the experiment shown in Figure 9*D*, no major change in absorbance was seen from 4 to 60 s (data not presented). *k*_on_ measurements were not done because of the need to complex sterols to 2-hydroxypropyl-β-cyclodextrin (HPCD) for solution, and the release rates are unknown.

### Single-turnover kinetics of oxidation of dihydrolanosterol

A powerful approach to studying the processivity of multistep reactions is a so-called “single-turnover” experiment, which works well if there is a high substrate affinity (43). Such an experiment requires rapid-quench kinetics and has been applied in cases of P450s 2E1 (9, 44), 19A1 (11), 17A1 (12), and 11B2 (13).

**Figure 10:**
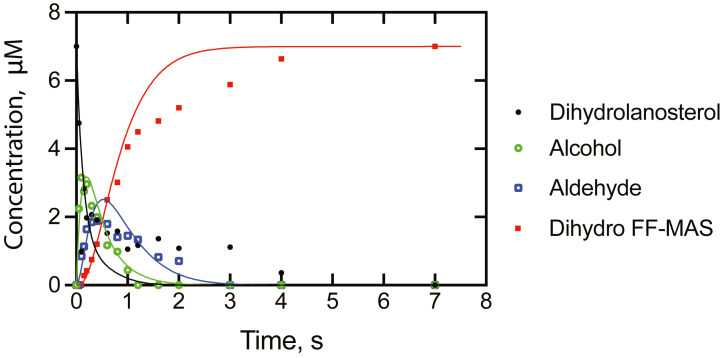
**Single-turnover time course of the reaction of a complex of 7 μM P450 51A1 and 7 μM [3-**^**3**^**H]-dihydrolanosterol, initiated by the addition of NADPH.** At each of the indicated times, the reaction was quenched and the individual products were extracted and quantitated using radio-HPLC as described in the Experimental procedures. The data points are shown for these individual products. The traces are shown for a model using the rate constants presented in Table 1. In each case, the total concentration of free and enzyme-bound compound was used. Dihydrolanosterol (●—●, *black line*); 14α-CH_2_OH dihydrolanosterol (, *green line*); 14α-CHO dihydrolanosterol (, *blue line*); and dihydro FF-MAS (, *red line*).

A 14 μM complex of P450 51A1- [3-^3^H]-dihydrolanosterol (Fig. S20) (plus phospholipid and 28 μM NADPH-P450 reductase) was diluted two-fold by rapid mixing with an NADPH solution and the reaction was then quenched with HCl at varying times. All products were separated and quantified by radio-HPLC (Fig. S24). The reaction was completed in 7 s, and the sequential oxidation of dihydrolanosterol to 14α-CH_2_OH dihydrolanosterol, 14α-CHO dihydrolanosterol, and dihydro FF-MAS was observed (Figs. 10 and S25). The observed order of production of the individual products was that shown in Figure 1, that is, dihydrolanosterol → 14α-alchohol → 14α-aldehyde → dihydro FF-MAS.

### Kinetic modeling

**Figure 11:**
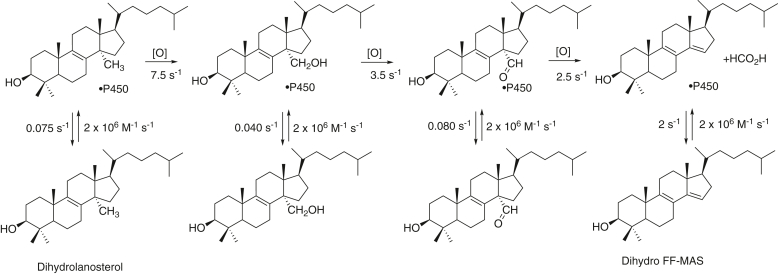
**Three-step oxidation of dihydrolanosterol to FF-MAS with optimized rate constants.** See Table 1 and Fig. S25. FF-MAS, follicular fluid meiosis-activating sterol ((4β,5α)-4,4-dimethyl-cholesta-8,14,24-trien-3-ol).

A minimal kinetic model was set up to fit the data obtained in the single-turnover experiment (Figs. 10 and 11), also utilizing the *k*_cat_, *K*_d_, and *k*_off_ estimates. The model and parameters used for the best fit are shown in Table 1 (Fig. S25).

**Table 1:** Model and kinetic parameters (rate constants) used for fitting

Reaction step		*k*^+^	*k*^-^
E + S ⇄ ES	*k*_1_, *k*_-1_	2 × 10^6^ M^−1^ s^−1^	0.075 s^−1^
ES ➝ EP	*k*_2_, *k*_-2_	7.5 s^−1^	0
E + P ⇄ EP	*k*_3_, *k*_-3_	2 × 10^6^ M^−1^ s^−1^	0.040 s^−1^
EP ➝ EQ	*k*_4_, *k*_-4_	3.5 s^−1^	0
E + Q ⇄ EQ	*k*_5_, *k*_-5_	2 × 10^6^ M^−1^ s^−1^	0.082 s^−1^
EQ ➝ ER	*k*_6_, *k*_-6_	2.5 s^−1^	0
E + R ⇄ ER	*k*_7_, *k*_-7_	2 × 10^6^ M^−1^ s^−1^	2.0 s^−1^

E: P450 51A1, S: dihydrolanosterol, P: 14α-CH_2_OH dihydrolanosterol, Q: 14α-CHO dihydrolanosterol, R: dihydro FF-MAS.

All *k*_on_ rate constants were set at 2 × 10^6^ M^−1^ s^−1^, a value similar to that used in our other P450 studies (11, 12, 13, 45, 46, 47) and consistent with an observed rate of 25 s^−1^ with 10 μM ketoconazole. The initial *k*_off_ rate constants used in fitting were the *k*_off_ rates measured in the stopped-flow trap experiments (Fig. 9). All three oxidation reactions are irreversible, and thus *k*_-2_, *k*_-4_, and *k*_-6_ were set to zero. The decrease in the concentration of dihydrolanosterol fit a first-order exponential decay with a rate of 8.7 s^−1^ (note 50% decrease of substrate concentration by 0.10 s), and this value was used as the rate constant *k*_2_ in the initial fitting. Thus, the only remaining rate constants needed to initiate fitting were *k*_4_ and *k*_6_, and initial 2-exponential fits of the data in KinTek Explorer yielded respective values of 2.1 and 2.2 s^−1^, which were used. The model was further optimized by visual approximation to generate the rate constants listed in Table 1 (fits in Figs. 10 and S25). Use of these parameters in models using the steady-state conditions gave *k*_cat_ values that exceeded those measured experimentally ∼1.5-fold, except for the aldehyde as substrate (Fig. 7). The *k*_on_ rate constants did not matter very much, and the principal drivers in fitting were the rate constants for the oxidations (*k*_2_, *k*_4_, and *k*_6_) and two of the *k*_off_ rate constants (*k*_-3_ and *k*_-5_), which have an experimental basis (Fig. 9).

Fitting of the overall oxidation of dihydrolanosterol to dihydro FF-MAS to a single exponential gave an apparent rate of 0.8 s^−1^, even with the lag, faster than the *k*_cat_ measured for the reaction (0.50 s^−1^, Fig. 7*A*).

One anomaly is the persistence of the initial substrate, dihydrolanosterol, in that ∼20% of the initial material persisted until > 4 s, even though the levels of the 14-alcohol and 14-aldehyde had decreased to zero (Fig. 10). A corresponding gap in dihydro FF-MAS production was also seen between 1.5 and 3.5 s (Fig. 10). This pattern was repeatable in a separate experiment (Fig. S25*A*). It does not appear to be the result of excess dihydrolanosterol compared to P450 51A1, and modeling such a scenario did not resolve the discrepancy. The behavior, in principle, could be explained by a conformation of the enzyme that oxidizes dihydrolanosterol at a slower rate, but such a possibility would be very difficult to prove and it has not been added to the model.

## Discussion

The synthetic procedure described here (Figs. 3 and S1) yielded reagents that could be used to address several outstanding questions. The intermediate 14α-alcohol and 14α-aldehyde were used as substrates (and products) in kinetic analyses. The synthesis of dihydro FF-MAS allowed for the use of an LC-UV assay that could be conducted with nonradioactive substrates, *d*_3_-dihydrolanosterol was used to address the question of rate-limiting C-H bond cleavages, and epi-dihydrolanosterol was used to examine the necessity of the β-orientation of the 3-OH in the substrate molecules.

The results support a processive mechanism for the overall conversion of dihydrolanosterol to dihydro FF-MAS (Fig. 11), with the *k*_off_ rates of the initial substrate and intermediate products being ≤5% of the rates of the individual oxidations. These conclusions are based on the *K*_d_ determinations (Fig. 8) and relying even more on the observed *k*_off_ rates measured in trap experiments (Fig. 9) and fitting of the single-turnover time course to a minimal kinetic model (Fig. 10). Only very limited information about processivity of this reaction was available earlier. Shafiee *et al.* (18) examined rat liver microsomal systems (fortified with cyanide to prevent some other reactions of lanosterol) for 2 h and found accumulation of the 14α-alcohol and 14α-aldehyde in ∼ 3% yield, but this result does not directly address the issue of processivity. In our model, the change to steady-state conditions (*e.g.*, excess substrate) resulted in a simulated accumulation of 0.4% of the 14α-alcohol and 1% 14α-aldehyde (results not shown). Of course, lowering the rate constants for the three individual oxidation steps attenuated the apparent processivity.

We are unaware of previous efforts to identify rate-limiting steps in the P450 51A1 reactions. The lack of an observed kinetic deuterium isotope effect on the overall conversion of 14α-CD_3_-dihydrolanosterol rules out C-H breaking as rate-limiting step in the first two reactions (Fig. 1). (A C-15 C–H bond is broken in the third step, but we have not addressed that issue.) Our results also indicate that release of the final product (dihydro FF-MAS) is not rate-limiting in the overall sequence. Whether the rate of electron transfer (to the ferric iron atom or Fe^2+^O_2_ complex) is rate-limiting has not been investigated. However, it is useful to note the high rate of hydroxylation of dihydrolanosterol in the first step of the human P450 51A1–catalyzed 14α-demethylation reaction (Fig. 10, ∼7 s^−1^ or 420 min^−1^), which is one of the fastest oxidation reactions catalyzed by a mammalian P450 (46). Because the reaction was initiated by mixing with NADPH, the rate includes binding of the NADPH to NADPH-P450 reductase, flavin reduction, input of one electron to the P450, O_2_ binding, input of another electron, rearrangement to make Compound I, and hydrogen abstraction and oxygen rebound. We do not have a *K*_m_ value for the first step, but *k*_obs_/*K*_d_ can be estimated as 7 s^−1^/0.23 μM = 3 × 10^7^ M^−1^ s^−1^ (Figs. 8 and 10)

epi-Dihydrolanosterol, which appears to be an obscure natural product (32), was recovered as a side product in one of the synthetic steps and shown to bind to human P450 51A1 tightly and be demethylated at a similar rate as the common β-hydroxy isomer (Figs 5*B* and 7*A*), behavior similar to that earlier reported for the yeast (*Saccharomyces cerevisiae*) enzyme (34). An H-bond between the sterol C3-OH and the main chain oxygen of a P450 51 residue preceding the beginning of the β1-4 strand is observed in four P450 51 structures in the Protein Data Bank: *Trypanosoma brucei* [3P99] (48), *Trypanosoma cruzi* [6FMO] (49), human [6UEZ] (19), plus a sterol-forming bacterium, *Methylococcus capsulatus* [7SNM] (50). Whether this interaction is replaced by another with epi-lanosterol is presently a point of conjecture and future research. We also dealt with (dihydro) agnosterol, a common contaminant of lanosterol derived from natural sources (and also formed in our synthesis beginning with 7-dehydrocholesterol (Figs. 3 and S1)), apparently at the *m*-chloroperoxybenzoic acid oxidation step (step 7). The degree of contamination by dihydroagnosterol was only ∼2%, and studies with the material purified by preparative HPLC showed only weak inhibition of dihydrolanosterol 14-demethylation (IC_50_ ≥ 10 μM when using a substrate concentration of 25 μM).

The finding that dihydroagnosterol is a substrate for human P450 51A1 (Fig. 6) was somewhat surprising in light of its Δ^7,9^ desaturation (Fig. 2). Although we have not synthesized and tested the Δ^7^ analog of dihydrolanosterol as a substrate with human P450 51A1, the efficiency of rat P450 51A1 with the Δ^7^ analog was only ∼1% that of the natural Δ^8^ isomer (51, 52).

The processivity of human P450 51A1 14α-demethylation can be compared with other multistep P450s. P450 19A1, the three-step steroid aromatase, is a very distributive enzyme (11). P450s 2E1 and 2A6 appear to be rather processive enzymes, at least in the oxidations of ethanol (9) and short-chain *N*,*N*-dialkylnitrosamines (10, 53), although the kinetics have not been studied in as much detail. P450 11B2 is a processive enzyme, catalyzing the oxidation of deoxycorticosterone to aldosterone, although the kinetic scheme includes a relatively irreversible nonenzymatic conversion of one product (18-hydrocorticosterone) to a ketol (13). The oxidations of progesterone and pregnenolone by human P450 17A1 are partially processive (12). P450s 11A1, 24A1, and 27A1 also catalyze multistep oxidations (8) but only limited information is available regarding this processivity (54). In addition, many oxidations of drugs show multistep reactions but their processivity is unknown.

**Figure 12:**
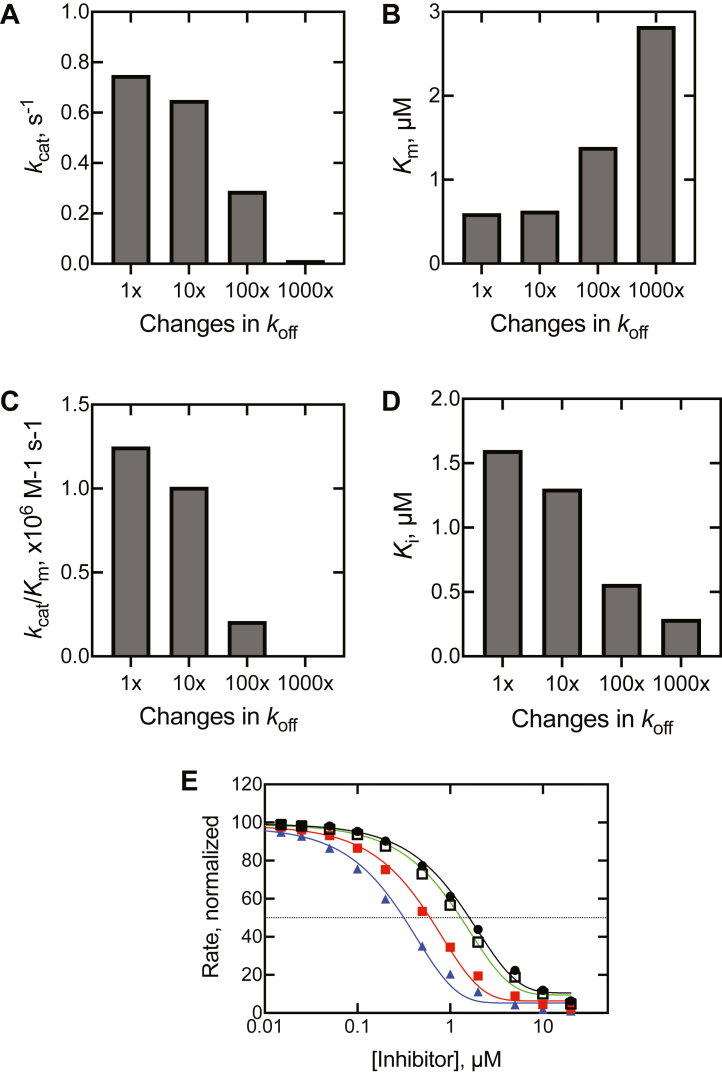
**Modeling of the conversion of dihydrolanosterol to dihydro FF-MAS and effects of changing only two *k***_**off**_**rate constants.** The rate constants for the dissociation of 14α-CH_2_OH dihydrolanosterol and 14α-CHO dihydrolanosterol (Table 1) were increased 10-, 100-, and 1000-fold, keeping all other rate constants constant (Table 1). The predicted changes are shown: *A*, *k*_cat_; *B*, *K*_m_; *C*, *k*_cat_/*K*_m_; *D*, *K*_i_ for an inhibitor included with a *K*_d_ of 25 nM. *E*, the predicted effect of a *K*_d_ 25 nM inhibitor is also shown in an IC_50_ plot (using 10 μM dihydrolanosterol) (*black line*, ●, 1 × ; *green line*, □, 10 × ; *red line*, ■, 100 × ; *blue line*, ▲, −1000 × ). The respective modeled IC_50_ values were 2.60, 1.17, 1.10, and 0.95 μM. FF-MAS, follicular fluid meiosis-activating sterol ((4β,5α)-4,4-dimethyl-cholesta-8,14,24-trien-3-ol).

The processivity of reactions has practical applications. In the presence of a high concentration of substrate, a processive enzyme should be less susceptible to enzyme inhibition, which can be readily demonstrated by changing *k*_off_ values in the presence of an inhibitor in simulations done with our model (Table 1, Fig. 12). Also, in the identification of pathways of drug metabolism (55), processive reactions can lead to products that might not be as readily identified in single oxidation queries. The biochemical relevance of processivity can be considered in quantitative terms, for example, modeling in Figure 12. Changing only the *k*_off_ rates of 14α-CH_2_OH dihydrolanosterol and 14α-CHO dihydrolanosterol has dramatic effects on the conversion of dihydrolanosterol to dihydro FF-MAS. Raising the two rate constants 1000-fold (to *K*_d_ values of 20 and 40 μM, Table 1) caused a > 500-fold decrease in *k*_cat_/*K*_m_ (Fig. 12), largely due to the change in *k*_cat_. The 1000-fold increase in the two *k*_off_ values also had the effect of increasing the sensitivity to an (undefined) inhibitor (modeled with *K*_d_ 25 nM), which might seem intuitive, in which the enzyme would be available for binding the inhibitor most of the time. The effect of a 1000-fold increase on the two *k*_off_ values on *K*_i_ and IC_50_ was only ∼5-fold (Fig. 12, *D* and *E*), however, much less remarkable than the >500-fold decrease in *k*_cat_/*K*_m_ (Fig. 12*C*).

In some cases, the processivity of P450 reactions can be rationalized in the context of the physiological relevance of the intermediates, that is, whether the intermediate is required as a substrate for an entirely different reaction. In the present case, there is no clear secondary role of the 14α-alcohol or 14α-aldehyde intermediates of lanosterol demethylation. Consequently, processivity of the P450 51 oxidation is efficient biologically in that it drives the reaction to completion, releasing a product that goes on to fulfill a vital role in cholesterol biosynthesis. In certain P450 reactions, however, the intermediate is a critical substrate of an entirely different pathway, presenting a potential biological requirement for a distributive oxidative mechanism. An example is the two-step oxidation of progesterone and pregnenolone by P450 17A1, wherein both the intermediate (17α-hydroxysteroid) and product (androgen) have critical biological roles (in the biosynthesis of glucocorticoids and androgens, respectively). In this case, a highly processive mechanism would preclude the biosynthesis of critical glucocorticoids, as the 17α-hydroxysteroid intermediate would not be released from the enzyme to be subsequently bound by other enzymes. The finding of both processive and distributive (“mixed”) character in this oxidation (12) then makes biological sense, as the enzyme mechanistically balances the biological need for both androgens (processive mechanism) and glucocorticoids (distributive mechanism) *via* partial release of the 17-hydroxysteroid. In this way, the physiological relevance of the intermediate(s) of multistep reactions may provide a physiological explanation for processive *versus* distributive character in some P450 steroid oxidations.

Another teleological reason for high processivity could be the protection of reactive intermediates along the reaction pathway. In this case, the 14α-aldehyde could be considered a potentially unstable compound, although we did not encounter any noticeable problems in the synthesis and handling of this molecule (Fig. S16). Processive enzymes also afford opportunities of yielding novel chemical entities for drug discovery efforts.

In conclusion, human P450 51A1 was shown to be a highly processive enzyme, with limited accumulation or release of intermediate products. Whether this is the situation in microbial and P450 family 51 enzymes is currently unknown but can be addressed using the approaches developed here.

## Experimental procedures

### Enzymes

Human P450 51A1 was expressed in *Escherichia coli* and purified as described (20, 56). Rat NADPH-P450 reductase (POR) was also expressed in *E. coli* and purified as described (57).

P450 concentrations were estimated spectrally using the extinction coefficient of 91,000 M^−1^ cm^−1^ for the ferrous–CO complex *versus* ferrous P450 difference spectrum (58, 59).

### Reagents

Crude lanosterol was purchased from Ambeed (Lot #A371706-008). Lanosterol and NaB^3^H_4_ were purchased from American Radiolabeled Chemicals. In general, chemicals were purchased from Millipore-Sigma-Aldrich or Thermo Fisher Scientific unless stated otherwise. Concentrations were based on dry weights used to dissolve materials.

### Synthesis

See Supporting Information section.

### Preparation of sterol solutions

When necessary, the studied sterols were purified prior to use in recombinant enzyme incubations. Sterol solutions (10 mM in C_2_H_5_OH) were applied to a Beckman Ultrasphere octadecylsilane (C_18_) semipreparative (10 mm × 250 mm, 5 μm) HPLC column using a mobile phase composed of CH_3_CN (100%) at a flow rate of 5 ml min^−1^. Peaks corresponding to the desired sterol were identified by their UV traces and were collected in fractions (∼5 ml), which were pooled and concentrated to dryness *in vacuo* (rotary evaporation, 80 mbar, 32 °C). Each dried sterol was collected in CH_2_Cl_2_ (3 × 2 ml rinses, pooled), and the sample was brought to dryness under a stream of N_2_ in a glass vial. The dried residue was then dissolved in C_2_H_5_OH (to 10 mM), sonicated until dissolved, and was then reinjected on HPLC to verify purity. The C_2_H_5_OH stock was then diluted 20-fold in HPCD solution (45% w/v) to 500 μM, which became the working stock concentration for all experiments with recombinant P450 51A1.

### UV-visible spectroscopy

Spectra were recorded using either an OLIS-Cary 14 or OLIS-Aminco DW2a instrument (On-Line Instrument Systems) in the split-beam mode.

### NMR spectroscopy

NMR spectra were recorded in CDCl_3_ on Bruker AV-400 or AV-II-600 instruments operating at 400.13 or 600.13 MHz in the Vanderbilt University Small Molecule NMR Facility Core.

### Liquid chromatography-mass spectrometry

LC-MS data were collected in the Vanderbilt Mass Spectrometry Research Center using Waters ACQUITY ultra-performance liquid chromatography (UPLC) Systems connected to Thermo Fisher Scientific LTQ XL Orbitrap mass spectrometers operating in the atmospheric pressure chemical ionization mode. Details are described in the Supporting Information.

### LC-UV assays

Steady-state incubations (500 μl) were generally done at 37 °C with 0.025 μM P450 51A1, 0.10 μM NADPH-P450 reductase, 100 μM L-α-dilauroyl-*sn*-glycero-3-phosphocholine, 50 mM potassium phosphate buffer (pH 7.4), 10% (v/v) glycerol, and an indicated concentration of each sterol (in 45% (w/v) HPCD). Due to the high turnover rate of the 14α-aldehyde, the P450 and NADPH-P450 reductase concentrations were reduced (to 0.01 μM and 0.04 μM, respectively), and the reaction volume was increased (to 1 ml) to maximize sensitivity. The sterols (stored at 4 °C) were prepared as described above and were heated to 37 °C and sonicated prior to use. Sterol working stocks were diluted in HPCD to ensure a constant concentration of cyclodextrin in each reaction. We established that the presence of 10% (v/v) glycerol (19) did not change the rates of reaction but did yield much greater consistency in the results, presumably due to better solubility of the sterol-HPCD. Accordingly, all reactions were done with this concentration of glycerol.

Reactions were preincubated (5 min, 37 °C) prior to initiation with an NADPH-regenerating system (0.5 mM NADP^+^, 10 mM glucose 6-phosphate, and 2 μg ml^−1^ glucose 6-phosphate dehydrogenase) (60) and were stopped after 5 min with the addition of CH_2_Cl_2_ (5 ml). (The NADPH-generating system, in addition to lower cost than NADPH, drives the reaction to avoid inhibition of the reductase by accumulated NADP^+^). For the 14α-aldehyde, the times of preincubation and reaction were reduced (to 2 min and 1 min, respectively). The quenched mixture was centrifuged (2000*g*, 5 min), and 4 ml of the bottom (organic) layer was transferred to vials and brought to dryness under a stream of N_2_ gas. The dried residue was resuspended in CH_3_OH (100 μl) and was analyzed by UPLC using a 2.1 mm × 100 mm (1.7 μm) octadecylsilane (C_18_) column (held at 25 °C) and a Waters Acquity UPLC system. Samples (4 °C) were injected (10 μl) at a flow rate of 0.20 ml min^−1^ using an isocratic mobile phase of CH_3_CN (only). The product (FF-MAS) was detected using a Waters Acquity photodiode array detector set at 250 nm (Fig. 4*B*). Data were processed using the MassLynx software (http://www.waters.com/waters/en_US/MassLynx-Mass-Spectrometry-Software-/nav.htm?cid=513164&locale=en_US), and the amount of product formed was calculated by comparison to a twelve-point dihydro FF-MAS standard curve. Determination of k_cat_ and K_m_ for each reaction was carried out by fitting the data to a Michaelis–Menten hyperbola, which was done using GraphPad Prism.

Incubations were run in triplicate, and the mean values (±SD) were calculated and plotted. The linear regression fits include the Prism error estimates for internal fitting (SE).

### Estimation of K_d_ for HPCD–dihydrolanosterol complex

The general method of Mast *et al.* (61) was used, in which 400 μM concentrations of dihydrolanosterol were shaken for 16 h with varying concentrations (0.5–94 mM) of HPCD in 100 mM potassium phosphate buffer (pH 7.4) containing 2% C_2_H_5_OH (v/v) (needed to dissolve the dihydrolanosterol stock at 20 mM (62)). After 16 h of vigorous shaking (at 23 °C), OD_450_ values were read and the (decreased) values (due to reduced light scattering) were measured in an OLIS-Cary 14 spectrophotometer. The OD_450_ values were fit to hyperbolae. Two assays yielded *K*_d_ values of 6.4 and 6.8 mM.

The highest concentration of HPCD used in catalytic assays and spectral *K*_d_ determinations was 15.5 mM, which is higher than the K_d_, but the critical points near the *K*_d_ value were much lower in HPCD (Figs. 5*A* and 8). Accordingly, the estimated *K*_d_ and *K*_m_ values for the sterol–P450 51A1 complexes were not corrected because of the low contribution of the HPCD under these conditions.

### K_d_ estimates

*K*_d_ values were estimated by difference spectroscopy. P450 51A1 (1.0 μM) was in each of two glass cuvettes (in 100 mM potassium phosphate buffer, pH 7.4) and a baseline was recorded (OLIS-Amino DW2a). Aliquots of a solution of each sterol–HPCD complex were added to the sample cuvette and an equivalent amount of HPCD was added to the reference cuvette, and spectra were recorded. The time between the collection of individual spectra was ∼3 min. The maximum absorbance difference (usually Δ*A*_390_-*A*_418_) was plotted *versus* the nominal concentration of added sterol, and the data were fit to hyperbolae in GraphPad Prism software (GraphPad, https://www.graphpad.com), using a quadratic equation to correct the ligand concentration for the enzyme-bound concentration (used as Y=B+(A/2)∗(1/E)∗((Kd+E+X)-sqrt((Kd + E+X)^∧^2-(4∗E∗X)) in Prism software). The linear regression fits include the internal error estimates (SE).

### k_off_ estimates

All assays were done at 23 °C in an OLIS-RSM 1000 stopped-flow spectrophotometer, collecting data every 1 ms over a range of 332 to 565 nm with slit widths of 1.24 mm (8 nm bandpass) and gratings with 400 lines/mm and 500 nm blaze. One syringe contained 20 μM ketoconazole dissolved in 100 mM potassium phosphate buffer (pH 7.4). In a preliminary experiment in which this was mixed with an equal volume of 2 μM P450 51A1 (same buffer), the observed rate of binding (Δ*A*_430_) was 25 s^−1^.

P450 51A1 (2 μM) was premixed with an equivalent concentration of each of the sterols (in HPCD). Each of these solutions was placed in a drive syringe and rapidly mixed with 20 μM ketoconazole (yielding final concentrations of 1 μM P450 51A1 and 10 μM ketoconazole). The rate of the Δ*A*_430_-*A*_390_ change was used as the *k*_off_ rate, that is, the rate at which the sterol left the P450 and allowed it to bind to the trap ligand ketoconazole.

### HPLC-radioactivity assays

Samples (held at 4 °C) were injected (45 μl) onto a Nova-Pak 3.9 mm × 150 mm (4 μm) octadecylsilane (C_18_) HPLC column (held at 23 °C). The products were separated at a flow rate of 1.0 ml min^−1^ using an isocratic mobile phase composed of CH_3_CN (only) as was done for the LC-UV assays (above). The eluate from the column was mixed (1:2, v-v) with FlowLogic U scintillation cocktail, and radioactivity (^3^H) was detected on a β-RAM Model 5 system (IN/US, LabLogic) (Fig. S24). Product formation was assessed as the relative contribution of each peak (in CPM) to the summed peak area of substrate and product(s).

### Single-turnover rapid-quench assays

The single-turnover experiments were performed using a KinTek rapid quench-flow apparatus (KinTek). This machine functions by forcing equal volumes (∼19 μl) of two separate solutions into a central mixing chamber, where the components are allowed to incubate for a predetermined period of time before an excess volume of quench solution (∼160 μl) stops the reaction and expels the mixture from the apparatus. Reactions (∼38 μl) were carried out by mixing equal volumes of a solution of 7 μM P450 51A1, 7 μM [3-^3^H]-dihydrolanosterol (7.55 mCi/mmol), 14 μM NADPH-P450 reductase, 200 μM L-α-dilauroyl-*sn*-glycero-3-phosphocholine, 10% (v/v) glycerol, and 50 mM potassium phosphate (pH 7.4) with a solution of 8 mM NADPH prepared in identical concentrations of potassium phosphate and glycerol. After the predetermined incubation period, the reaction (37 °C) was quenched with HCl (1 M), and the mixture (∼200 μl) was collected into vials. The products of five identical reactions (time points) were combined and were extracted into CH_2_Cl_2_ (5 ml). An aliquot (4 ml) of the lower (organic) layer was removed into a fresh vial, and the extraction was repeated to maximize sample recovery. The pooled organic fraction (∼8 ml) was brought to dryness under a stream of N_2_ gas, and the residue was redissolved in 60 μl of CH_3_OH and transferred to LC vials for analysis.

### Kinetic modeling

Data were imported into KinTek Explorer software (v. 11.01, KinTek, https://kintekcorp.com/software) (63, 64) as txt files and processed using an Apple computer (operating system 11.6.2).

## Data availability

All data are available in the Supporting Information in the form of synthetic procedures and characterization of chemicals, plus NMR, mass, and UV spectra. Also, key txt files include the data used for calculations or *k*_cat_, *K*_m_, and *K*_d_ and fitting to kinetic models.

## Supporting information

This article contains supporting information, (21, 22, 23, 28, 65, 66, 67).

## Conflict of interest

All of the authors declare that they have no conflict of interest with the contents of this article.
