# Supplementary material for: Processive kinetics in the three-step lanosterol 14α-demethylation reaction catalyzed by human cytochrome P450 51A1
Source: J Biol Chem. 2023 May 18;299(7):104841. doi: 10.1016/j.jbc.2023.104841 (PMC10285260; doi:10.1016/j.jbc.2023.104841)
Supplement: Supporting Table S1 and Figures S1–S25 [file mmc1.docx]

**Supporting Information**

**Processive kinetics in the three-step lanosterol 14α-demethylation reaction catalyzed by human cytochrome P450 51A1**

Kevin D. McCarty, Molly E. Sullivan, Yasuhiro Tateishi, Tatiana Y. Hargrove, Galina I. Lepesheva, and F. Peter Guengerich

Department of Biochemistry, Vanderbilt University School of Medicine, Nashville, Tennessee 37232-0146. United States

**Table of contents**

**Synthetic procedures**  p. S-3

**Reagents** p. S-3

**General** p. S-3

**Dihydrolanosterol** p. S-3

**Synthesis of 24,25-dihydrolanosterol 14α-alcohol and 14α-aldehyde** p. S-4

**Step 1.** p. S-4

**Step 2.** p. S-5

**Step 3.** p. S-5

**Step 3.** p. S-5

**Step 4.** p. S-5

**Step 5.**  p. S-5

**Step 6.** p. S-5

**Step 7.** p. S-6

**Step 8.** p. S-6

**Step 9.** p. S-6

**Step 10.** p. S-6

**Step 11.** p. S-7

**Step 12.**  p. S-12

**Step 13.** p. S-12

**Step 14.**  p. S-20

**Step 15.**  p. S-21

**Step 16.**  p. S-21

**24,25-Dihydro FF-MAS**  p. S-22

**[3-^3^H]-Dihydrolanosterol** p. S-24

**[32-^2^H]-Dihydrolanosterol** **(*d*_3_)** p. S-25

**epi-Dihydrolanosterol** p. S-26

**Agnosterol** p. S-26

**Kinetic analysis** p. S-28

**Txt files for analysis** p. S-28

***k*_cat_, *K*_m_ determinations (for FF-MAS formation)** p. S-28

***K*_d_ determinations** p. S-29

**Single turnover kinetics concentration files** p. S-30

**Radio-HPLC trace** p. S-30

**Screen shots for KinTek Explorer fitting** p. S-31

**Synthetic procedures**

**Reagents**. 7-Dehydrocholesterol (> 90% purity) and most of the reagents were purchased from Sigma-Aldrich-Millipore. Benzyl chloromethyl ether was purchased from TCI America, and lanosterol and NaB^3^H_4_ were purchased from American Radiolabeled Chemicals. Solvents and commercial reagents were used without further purification.

**General**. NMR spectra were recorded on Bruker AV-400 or AV-II-600 instruments in the Vanderbilt University Small Molecule NMR Facility Core, operating at 400.13 or 600.13 MHz. Tetramethylsilane (TMS, δ=0.00 ppm) was used as an internal standard for ^1^H experiments, and an NMR solvent signal (δ=77.16 ppm for CDCl_3_) was used as a reference for ^13^C experiments.

All mass spectra of sterols were collected with high resolution mass spectrometry (HRMS) using an LTQ XL Orbitrap instrument (Thermo Fisher) in the APCI (positive ion) mode in the Vanderbilt Mass Spectrometry Core Facility, after introduction from UPLC using a Waters ACQUITY system equipped with a ZORBAX Rx-C8 column (5 µm, 2.1 mm × 150 mm) (Agilent). Chromatographic separation was conducted at 0.2 ml min^-1^ flow rate using isocratic elution of CH_3_CN containing 0.1% (v/v) HCO_2_H. For HRMS, the Orbitrap instrument was set to full scan mode, FTMS analyzer, 60,000 resolution, and scanned *m/z* from 100 to 1000. The APCI source was adjusted as follows: vaporizer temperature 350 °C, capillary temperature 275 °C, capillary voltage 48 V, and tube lens 125 V.

UV spectra were recorded either on-line from UPLC using a Waters Acquity instrument (diode array) or using an OLIS-Cary 14 or an OLIS-Aminco DW2a spectrophotometer (On-Line Instrument Systems, Athens, GA) (in C_2_H_5_OH).

Silicic acid chromatography was done using columns of SiliFlash F60 (230-400 mesh). Analytical TLC was done with SilicaGel 60 F_254_ plates and preparative TLC with 2 mm Silica Gel 60 F_254_ plates (both from Merck KGaA), with mixtures of hexanes and ethyl acetate, with visualization by UV light (254 nm) and/or spraying with phosphomolybdic acid solution and heating.

**Dihydrolanosterol**

Crude lanosterol was purchased from AmBeed and recrystallized from hot acetone and then from acetone/H_2_O. The preparation contained ~60% lanosterol and 40% 24,25-dihydrolanosterol (analysis by LC-MS). The material (5.8 g) was dissolved in 90 ml of a benzene-ethyl acetate mixture (1:1, v-v) and 1.25 g of Pd on carbon (10%) was added. Hydrogenation was done at 45 psi (3 atm) overnight at 23 °C. The product was filtered through Celite, which was washed with ethyl acetate. The eluted material was concentrated to dryness *in vacuo* and recrystallized from acetone.

**Synthesis of 24,25-dihydrolanosterol 14α-alcohol and 14α-aldehyde** (21-23)

**Figure S1. Synthesis of dihydrolanosterol with -CH_2_OH and -CHO at position 14.** BOM, benzoyl methyl ether; Dess Martin (periodinane reagent), 3-oxo-1λ^5^,2-benziodoxole-1,1,1(3*H*)-triyl triacetate; mCPBA, *m*-chloroperbenzoic acid; PCC, pyridinium chlorochromate; Pv, pivaloyl (trimethylacetyl);

**Step 1.** 7-Dehydrocholesterol (**1**) (30 g, 78 mmol) was heated at reflux (with stirring) in a mixture of 300 ml toluene, 60 ml cyclohexanone (580 mmol), and 8.4 g of aluminum isopropoxide (42 mmol) in a 1-l round bottom flask for 2 h; 60 ml of the solution was removed by use of a Dean-Stark trap after reflux began. The product (4,7 dien-3-one, **2**) was extracted into ethyl acetate, and the solution was sequentially washed 3× each with brine (saturated aq NaCl) and H_2_O. The ethyl acetate phase was dried with Na_2_SO_4_, filtered through paper, and concentrated *in vacuo*. The residue was dissolved in a minimum volume of CH_2_Cl_2_ and applied to a 7.5 cm × 32 cm silica column, which was washed with hexanes and then eluted with mixtures of hexanes-ethyl acetate (20:1, v/v) followed by a 10:1 hexanes/ethyl acetate mixture (v/v). Fractions (250 or 600 ml) were collected and monitored using TLC (hexanes-ethyl acetate, 9-1, v-v). The product (which eluted with the 10:1 hexanes/ethyl acetate mixture) was used directly in step 2.

**Step 2**. The 4,7-dien-3-one (**2**) from step 1 was dissolved in 35 ml of tetrahydrofuran and added by dropwise addition (over 40 min) to a stirred solution of 23.8 g (213 mmol) potassium *tert*-butoxide in 450 ml of *tert*-butanol in a 1-l round bottom flask. The solution turned a very dark red upon addition. CH_3_I (66 ml, 150 g, 1.1 mol) was then added dropwise, and the solution changed to a light brown opaque nature. Stirring continued for 30 min. The reaction was dissolved in hexanes and washed sequentially 3× each with brine, 5% aqueous HCl (w/v), and H_2_O. The resulting extract was dried with Na_2_SO_4_, filtered, and concentrated *in vacuo* to yield compound **3** (Fig. S1), HRMS (*m/z*) calcd for C_29_H_47_O, [M+H]^+^, 411.3621; found, 411.3608 (Δ -3.2 ppm).

**Step 3**. The 4,4-dimethyl product synthesized above (**3**) was dissolved in 300 ml of tetrahydrofuran. LiAlH_4_ (4.0 g, 0.105 mmol) was added (in aliquots) over 30 min. After addition was complete, the reaction was stirred for an additional 90 min (at 23 °C). The reaction was quenched by the sequential dropwise addition of 3 ml ethyl acetate, 4 ml H_2_O, 5 ml 3 M NaOH, and 4 ml H_2_O (65). The reaction was filtered through paper, and the residue was washed with ethyl acetate and then hexanes. The solvent was removed *in vacuo* and the residue was dissolved in hexanes and washed three times each with brine and then H_2_O. The solution was dried with Na_2_SO_4_, filtered, and concentrated *in vacuo* to give product **4**.

**Step 4**. Compound **4** was dissolved in 750 ml of ethyl acetate and 3.1 g of 10% Pd-carbon was added. The suspension was hydrogenated (20 psi, 1.5 atm) for 1 h in a Parr apparatus at 23 °C. The suspension was filtered *in vacuo* through a short bed of Celite, and the solvent was removed *in vacuo* to yield compound **5**: MS: *m/z* 397.4 (M+H -H_2_O) ^+^.

**Step 5**. The residue from preparation **5** was dissolved in 300 ml of CHCl_3_ and 16.3 ml *N*-ethyl-*N*,*N*-diisopropylamine (12.1 g, 94 mmol). The solution was stirred on ice and 11.6 ml (11.0 g, 91 mmol) of pivaloyl chloride (PvCl) was added over 30 min with an addition funnel (under Ar). The solution was stirred another 30 min at 23 °C and then heated under reflux for 3 h. (A prior trial using CH_2_Cl_2_ under reflux resulted in a poor yield of acylation.) The reaction was cooled and washed sequentially (3× each time) with brine, 5% HCl, H_2_O, saturated NaHCO_3_, and H_2_O. The CHCl_3_ layer was dried with Na_2_SO_4_, filtered, and concentrated to dryness *in vacuo*. The product (**6**) readily crystallized from hot acetone (total yield 13.9 g): mp 166-170 °C; HRMS (*m/z*) calcd for C_29_H_49_, [M+H-PvOH]^+^, 397.3829; found, 397.3811 (Δ -4.4 ppm);^1^H-NMR: δ 1.27 (s, 9H).

**Step 6**. A 2.5 g aliquot of **6** was dissolved in 500 ml of CH_2_Cl_2_ and cooled to -20 °C with the use of an ice-salt bath (3:1 ratio of ice/NaCl, w/w). The solution was sparged with HCl gas (from a lecture bottle) for 20 s, and the reaction was maintained at -20 °C for 15 min. (Previous kinetic trials in this lab had shown that this was the optimum time for isomerization to **7** (22)). NMR analysis of the product showed the expected vinyl proton (H-15) at δ 5.16. The H-3 peaks at δ 4.43 and 4.48 indicated that the product was a 2:1 mixture of **7** and **6**. These two isomers were not readily separable, and the mixture was used in the next step.

**Step 7**. The mixture of **7** and **6** (from above, nominally ~ 12.5 g) was dissolved in 300 ml of CH_2_Cl_2_ and *m*-chloroperbenzoic acid (1.25-fold nominal molar excess, 77% purity from Sigma-Aldrich-Millipore, 8.6 g, 39 mmol) was added in aliquots to come to 23 °C and stirred for another 2 h, when the mixture was washed sequentially three times each with saturated NaHCO_3_, brine, and H_2_O. The solution was dried with Na_2_SO_4_, filtered, and concentrated *in vacuo*.

**Step 8**. The product from the epoxidation reaction (**8**) was stirred in 200 ml of benzene at 23 °C and 5 ml of BF_3_·(C_2_H_5_)_2_O was added. After 15 min, CH_2_Cl_2_ was added and the solution was washed sequentially (3×) with saturated NaHCO_3_, brine, and H_2_O. The solution was dried with Na_2_SO_4_, filtered, and concentrated to dryness *in vacuo*. The residue was dissolved in CH_2_Cl_2_ and applied to a 7.5 cm × 30 cm silica column, which was washed with 1 l of hexanes and then sequentially with 3.2 l of hexanes/ethyl acetate (80:1, v/v), 4.8 l of hexanes/ethyl acetate (50:1, v/v), and 4 l of hexanes/ethyl acetate (10:1, v/v). 24,25-Dihydro FF-MAS (**19**) 3-pivaloyl ester (*vide infra*) eluted in the 50:1 solvent mixture and **9** eluted with the 10:1 solvent mixture. Both products were concentrated to dryness *in vacuo*.

**Step 9**. The material (**9**) from the above step (4.1 g, 7.9 nmol, in 20 ml CH_2_Cl_2_) was added to a stirred suspension of pyridinium chlorochromate (2.58 g, 12 mmol, 1.5-molar excess) in 60 ml of CH_2_Cl_2_. The reaction was stirred for 2 h at 23 °C (66) and then diluted with 300 ml of (C_2_H_5_)_2_O; the solvent was decanted and saved. The solid residue was washed with (C_2_H_5_)_2_O and added to the bulk solution, which was filtered through a 2.5 cm × 10 cm column of Florasil (60-100 mesh). The eluate was reduced *in vacuo*, taken up in CH_2_Cl_2_, washed 3× each with 5% HCl, brine, and H_2_O, followed by drying with Na_2_SO_4_, filtration, and concentration *in vacuo* to give 3.7 g of crude product (**10**): HRMS: (*m/z*) calcd for C_29_H_47_O, [M+H-PvOH]^+^, 411.3621; found, 411.3637 (Δ +3.9 ppm).

**Step 10**. Compound **10** (2.3 g, 4.5 mmol) was dissolved in 35 ml of a stirred mixture of 1.78 g potassium *tert*-butoxide (16 mmol) in *tert*-butanol. Chloromethyl benzyl ether (6.34 ml, 46 mmol) was added dropwise, and stirring was continued overnight. The solvent was reduced by one-half *in vacuo*, and the material was dissolved in (C_2_H_5_)_2_O. The (C_2_H_5_)_2_O solution was washed sequentially (3×) with H_2_O, 5% HCl, and H_2_O and then dried with MgSO_4_, filtered, and concentrated *in vacuo*. The crude product was applied to a 4.5 cm × 30 cm silica column, which was washed with 1 l hexanes, 1 l of 100:1 hexanes/ethyl acetate (v/v), 2.5 l of 50:1 hexanes/ethyl acetate, and 1.5 l of 25:1 hexanes/ethyl acetate (v/v). Compound **11** was eluted with 50:1 (v/v) hexanes/ethyl acetate: HRMS (*m/z*) calcd for C_42_H_63_O_3_, [M+H-H_2_O]^+^, 615.4772; found, 615.4771 (Δ -0.05 ppm); NMR (CDCl_3_) δ 3.37, 3.66 (2H, benzyl), 7.24-7.25 (5H, aryl).

**Step 11**. Compound **11** (from above) was dissolved in 60 ml of diethylene glycol, and 29 ml of hydrazine hydrate (64-65% NH_2_NH_2_, 590 mmol) was added (dropwise), in a 250-ml round bottom flask attached to a distilling head and water-cooled condenser, under an Ar atmosphere (balloon). The mixture was stirred at 160 °C for 4 h. After cooling, 24 g of KOH (62 mmol) was added, and the mixture was heated to 185 °C. About 20 ml of solvent was distilled off, and the temperature was raised to 210 °C, with vigorous evolution of N_2_ in the flask. Heating (and stirring) at 215 °C continued overnight, and the evolution of N_2_ had ceased. When the solution had cooled to ~ 100 °C, 50 ml of H_2_O was added and stirring continued for 30 min to complete hydrolysis of the pivaloyl ester. The solution was then added to CH_2_Cl_2_ and washed 3× each with brine, H_2_O, 5% aq. HCl, and H_2_O. The CH_2_Cl_2_ layer was dried with Na_2_SO_4_, filtered, and concentrated *in vacuo*. The residue was dissolved in a minimal volume of CH_2_Cl_2_ and applied to a 2.0 cm × 43 cm silica column, which was sequentially eluted with 120 ml hexanes, 500 ml hexanes/ethyl acetate (50:1, v/v), 500 ml hexanes/ethyl acetate (25:1, v/v), and 1000 ml hexanes/ethyl acetate (12:1, v/v). The product (**12**) eluted with the 12:1 hexanes/ethyl acetate mixture and the fractions containing only this compound (TLC) were pooled and concentrated *in vacuo*: HRMS (*m/z*) calcd for C_37_H_57_O, [M+H-H_2_O]^+^, 517.4404; found, 517.4419 (Δ +2.9 ppm); calcd for C_30_H_51_O, [M+H-BnOH]^+^, 427.3934; found, 427.3947 (Δ +3.0 ppm). For NMR assignments see Table S1 (^1^H,^13^C) and Figure S12 (NOESY).

**Figure S2. ^1^H NMR spectrum for compound 12.**

**Figure S3. ^13^C NMR spectrum for compound 12.**

**Figure S4. HSQC spectrum for compound 12.**

**Figure S5. HMBC spectrum for compound 12.**

**Figure S6. NOESY spectrum for compound 12.**

Compound **12**

^1^H NMR (600 MHz, CDCl_3_): δ 0.68 (s, H-18, 3H), 0.81 (s, H-28, 3H), 0.85-0.87 (m, H-21, 26, and 27, 9H), 0.95-0.97 (m, 1H), 0.99 (s, H-19, 3H), 1.01 (s, H-29, 3H), 1.08-1.19 (m, 5H), 1.33-1.38 (m, 5H), 1.43-1.49 (m, 2H), 1.50-1.55 (m, 2H), 1.56-1.60 (m, 1H), 1.64-1.68 (m, 4H), 1.69-1.73 (m, 1H), 1.74-1.78 (m, 1H), 1.92-1.96 (m, 1H), 1.98-2.02 (m, 2H), 2.04-2.09 (m, 1H), 2.21-2.28 (m, 1H), 3.25 (dd, *J*=11.6, 4.0 Hz, H-3, 1H), 3.30 (d, *J*=8.3 Hz, H-30, 1H), 3.40 (d, *J*=8.3 Hz, H-30, 1H), 4.37 (d, *J*=12.5 Hz, H-31, 1H), 4.52 (d, *J*=12.6 Hz, H-31, 1H), 7.23-7.26 (m, 1H), 7.28-7.33 (m, 4H).

^13^C NMR (150 MHz, CDCl_3_): δ 15.55 (C-29), 16.67 (C-18), 18.53 (C-6), 18.79 (C-21), 18.94 (C-19), 21.25 (C-11), 22.67 (C-26), 22.97 (C-27), 24.17 (C-23), 25.63 (C-15), 27.94 (C-2), 28.17 (C-28), 28.78 (C-16), 29.05 (C-7), 31.12 (C-12), 35.62 (C-1), 36.55 (C-22), 36.62 (C-20), 37.46 (C-10), 38.97 (C-4), 39.63 (C-24), 45.73 (C-13), 50.49 (C-5), 51.81 (C-17), 54.74 (C-14), 73.42 (C-31), 76.79 (C-30), 79.19 (C-3), 127.21 (C-33 and 35), 128.30 (C-34), 133.11 (C-8), 135.51 (C-9), 139.52 (C-32).

**Figure S7. ^1^H and ^13^C NMR assignments for compound 12.**

**Step 12**. Compound **13** (from above) was dissolved in 15 ml of CHCl_3_ and 0.52 ml (3.0 mmol) of *N*-ethyl-*N*,*N*-diisopropylamine and stirred on ice. Acetyl chloride (0.22 ml, 3.0 mmol) was added. The reaction (under Ar) was allowed to come to room temperature and then heated under reflux for 3 h, when TLC indicated that the reaction (to form **13**) was complete. The solution was cooled to room temperature and sequentially washed (3× each) with H_2_O, aq. 5% HCl, H_2_O, saturated (aq.) NaHCO_3_, and H_2_O. The CHCl_3_ layer was dried with Na_2_SO_4_, filtered, and concentrated to dryness *in vacuo*.

**Step 13**. The 3-acetyl ester (**13**) from step 12 was dissolved in 30 mL of C_2_H_5_OH and stirred with 37 mg of 10% Pd-C overnight under an H_2_ balloon. The suspension was filtered through a short bed of Celite, and the filtrate was concentrated to dryness *in vacuo* to yield 185 mg of the key intermediate **14**: HRMS (*m/z*) calcd for C_32_H_53_O_2_, [M+H-H_2_O]^+^, 469.4040; found, 469.4057 (Δ +3.6 ppm); calcd for C_30_H_49_, [M+H-H_2_O-HOAc]^+^, 409.3829; found, 469.3841 (Δ +3.1 ppm) (base peak). NMR: see Figs. S8, S9, S10, S11, S12, S13, and S14 and Table S1.

**Figure S8. ^1^H NMR spectrum for compound 14.**

**Figure S9. ^13^C NMR spectrum for compound 14.**

**Figure S10. HMBC spectrum for compound 14.**

**Figure S11. HSQC spectrum for compound 14.**

**Figure S12. NOESY spectrum for compound 14.**

Compound **14**

^1^H NMR (600 MHz, CDCl_3_) δ 0.70 (s, H-18, 3H), 0.862 (d, *J*=6.6 Hz, H-26, 3H), 0.867 (d, *J*=6.6 Hz, H-27, 3H), 0.88-0.90 (m, H-21, 28, and 29, 9H), 0.96-1.01 (m, 2H), 1.06 (s, H-19, 3H), 1.08-1.19 (m), 1.29-1.41 (m), 1.49-1.65 (m), 1.67-1.75 (m), 1.84 (dt, *J*=13.2, 3.4 Hz, 1H), 1.88-1.96 9m, 2H), 1.97-2.03 (m, 1H), 2.05 (s, -COCH_3_, 3H), 2.06-2.12 (m, 3H), 3.22 (d, *J*=11.0 Hz, H-30, 1H), 3.63 (d, *J*=11.0 Hz, H-30, 1H), 4.49 (dd, *J*=11.8, 4.4 Hz, H-3, 1H).

^13^C NMR (150 MHz, CDCl_3_) δ 16.59 (C-29), 17.49 (C-18), 18.07, 18.68(C-21), 19.17 (C-19), 21.31 (-COCH_3_), 21.91, 22.54 (C-26), 22.82 (C-27), 23.99, 24.11, 27.05 (C-15), 27.73, 27.94 (C-28), 28.01 (C-25), 28.17, 31.02 (C-12), 35.88, 36.31, 36.38, 37.51 (C-10), 37.87 (C-4), 39.50 (C-24), 45.06 (C-13), 50.42 (C-17), 50.99 (C-5), 55.87 (C-14), 66.66 (C-30), 80.69 (C-3), 129.84(C-8), 139.88 (C-9), 170.25 (-COCH_3_).

**Figure S13. ^1^H and ^13^C NMR assignments for compound 14.**

**Table S1.** **^1^H and ^13^C NMR assignments for compounds 12 and 14.** ^a^Corresponding proton or carbon does not exist; ^b^not assigned.

**
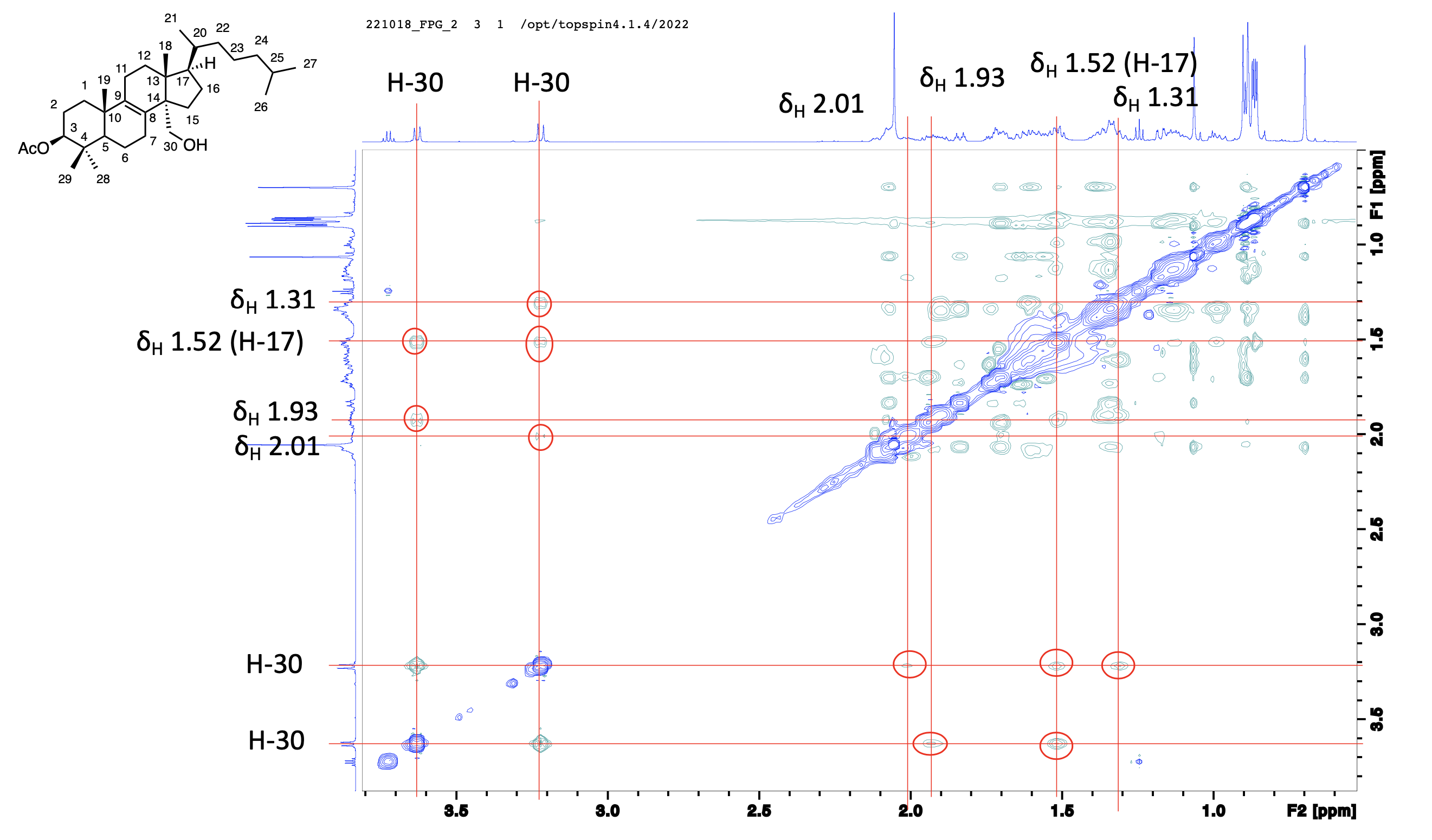
**

**Figure S14. Comparison of NOESY spectra of compounds 12 and 14**

**Step 14**. Compound **14** (93 mg, 0.19 mmol) was dissolved in 2 ml of CH_2_Cl_2_ and stirred with 85 mg of Dess-Martin periodinane (3-oxo-1λ^5^,2-benziodoxole-1,1,1(3*H*)-triyl triacetate) (67) (0.20 mmol) for 1 h at 23 °C, at which time TLC indicated that the reaction was complete. The reaction was diluted with 10 ml (C_2_H_5_)_2_O and added to 10 ml of saturated aq. NaHCO_3_ containing 220 mg (1.4 mmol) Na_2_S_2_O_3_. The (C_2_H_5_)_2_O layer was washed 3× with H_2_O and dried *in vacuo* to yield Compound **15**.

**Step 15**. Compound **14** (from step 13) was dissolved in 2 ml of benzene. To this was added 2 ml of CH_3_OH containing 2% (w/v) KOH. The mixture was stirred and heated at 60 °C for 4 h under Ar. After cooling, the mixture was added to 15 ml of (C_2_H_5_)_2_O, which was washed with brine and H_2_O. The product was concentrated *in vacuo* to yield the final product **16**. The residue (**16**) was concentrated to dryness *in vacuo*. HRMS (*m/z*) calcd for C_30_H_51_O, [M+H-H_2_O]^+^ 427.3934; found, 427.3920 (Δ -3.3 ppm); calcd for C_30_H_49_, [M+H-2H_2_O]^+^ 409.3829; found, 409.3815 (Δ -3.4 ppm)


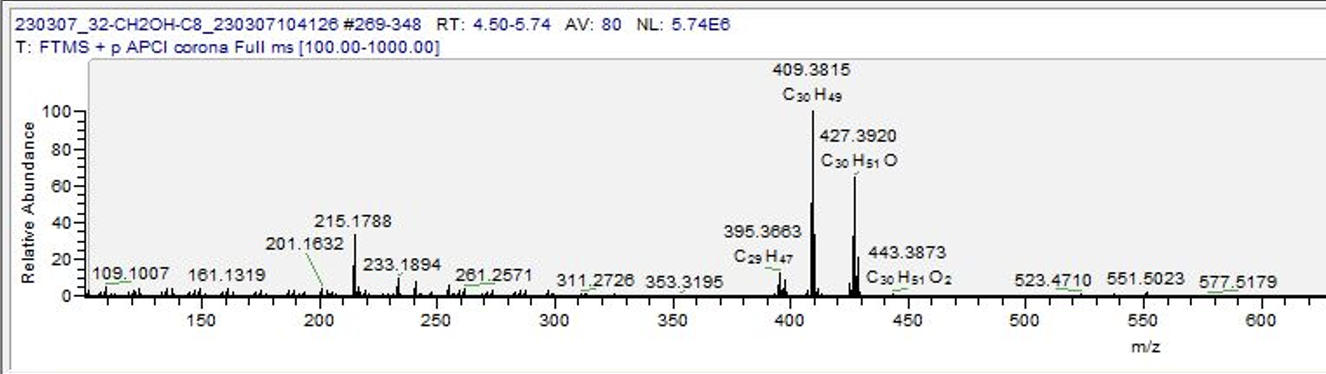


**Figure S15. HRMS mass spectrum of 24,25-dihydro-14α-CH_2_OH lanosterol (16).**

**Step 16**. Compound **15** (from step 14, not purified) was dissolved in 2 ml of benzene and hydrolyzed as in the case of step 15. The product was concentrated *in vacuo* to yield the final product **17**, the 14α-aldehyde (24,25-dihydro-14α-formyl lanosterol), which was purified by preparative HPLC prior to use in incubations. HRMS: (*m/z*) calcd for C_30_H_51_O_2_, [M+H]^+^ 443.3884; found, 443.33868 (Δ -3.6 ppm); calcd for C_30_H_49_O, [M+H-H_2_O]^+^ 425.3778; found, 425.3763 (Δ -3.5 ppm, base peak) (Fig. S16).


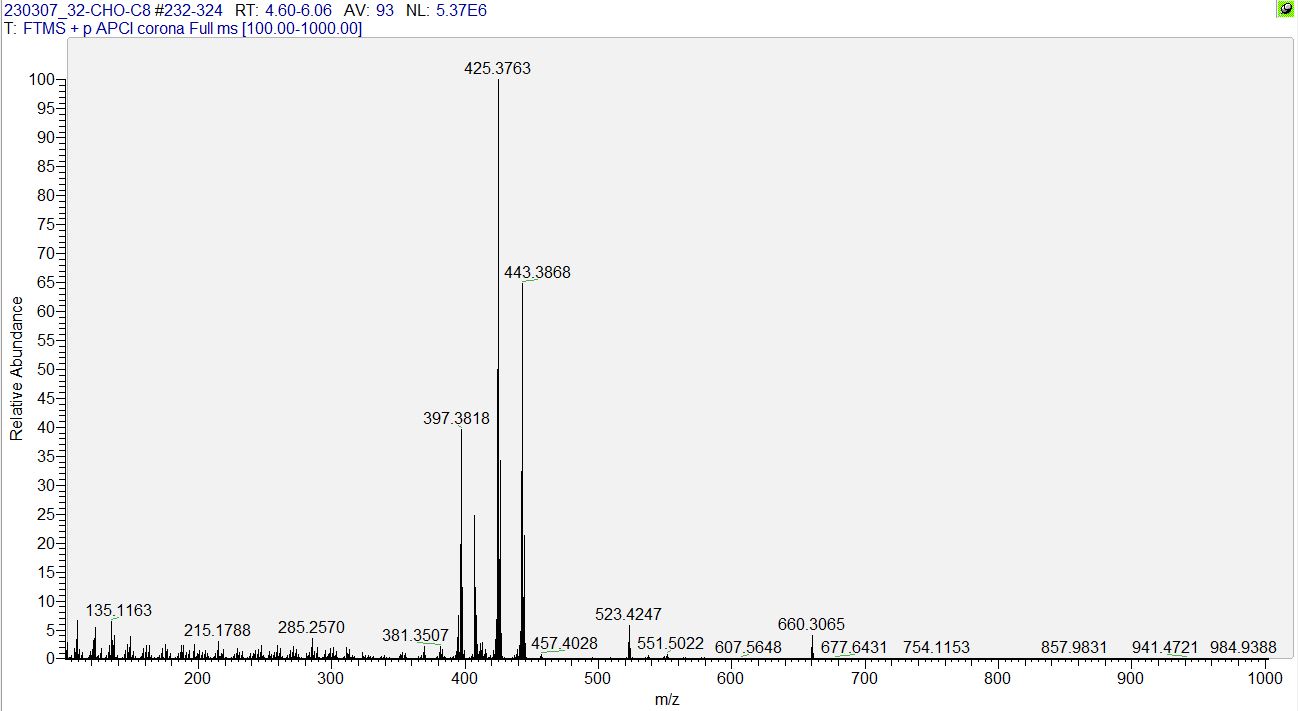


**Figure S16. HRMS mass spectrum of 24,25-dihydro-14α-formyl lanosterol (17).**

**Synthesis of 24,25-dihydro FF-MAS**

**Figure S17. Synthesis of 24,25-dihydro FF-MAS (19).**

Step 8 of the synthesis of **16** and **17** yielded a side product with a strong UV chromophore and higher mobility on silica chromatography. This compound (**18**) was recovered from the 50:1 hexanes/ethyl acetate (v/v) fraction of the column: *m/z* 497.4338 (C_34_H_57_O_2_, MH^+^), 395.3656 (C_29_H_47_, base peak, MH-PivOH^+^). Compound **18** resisted hydrolysis under the conditions used for acetate esters (*vide supra*) but the pivaloyl ester could be cleaved with LiAlH_4_ treatment (4-fold excess) in (C_2_H_5_)_2_O at room temperature (2 h). Quenching and extraction as in step 3 (Fig. S1) of the synthesis of **16** and **17** was done, and ½ of the resulting material was applied to a 2.0 cm × 45 cm silica column, which was washed with hexanes and then eluted with a mixture of hexanes/ethyl acetate (10:1, v/v). Compound **19** was eluted between 300 and 450 ml, as judged by analytical TLC (hexanes-ethyl acetate, 9-1, v/v). The combined fractions were pooled to give **19**: HRMS (*m/z*) calcd for C_29_H_47_, [M+H-H_2_O]^+^ 395.3672; found, 395.3663 (Δ -2.3 ppm); UV (C_2_H_5_OH) λ_max_ 250 nm (Fig. S18); NMR (CDCl_3_) δ 3.25 (H-3, dd), 5.36 (15-H). The NMR spectrum showed trace contamination with an H-3 peak (dd) at δ 3.30, which could not be removed by silica gel chromatography or preparative TLC. It was partially resolved by C_18_ (but not C_8_) HPLC; the UV and mass spectra was identical to the main component (**20**). The impurity is assigned as the 3α-hydroxy isomer of **20**, epi-dihydro FF-MAS. A possible explanation for its formation is presented in Fig. S19, if trace H_2_O was a contaminant.

**Figure S18. UV spectrum of 24,25-dihydro FF-MAS (Compound 20).** The concentration was 33 µM (in C_2_H_5_OH).

**Figure S19. Possible mechanism for presence of 3α-OH 24,25-dihydro FF-MAS.**

**[3-^3^H]-Dihydrolanosterol** (Fig. S20)

Dihydrolanosterol (*vide supra*) (82 mg, 0.20 mmol) was dissolved in 2 ml CH_2_Cl_2_ and stirred with (84 mg, 0.21 mmol) of Dess-Martin periodinane (3-oxo-Δλ^5^,2-benziodioxole-1,1,1(^3^*H*)-triyl triacetate) (67) for 1.5 h at 23 °C. Ethyl ether (10 ml) was added and the solvent was decanted and added to 10 ml of 10% aq NaHCO_3_ containing 221 mg of Na_2_S_2_O_3_ (7-fold molar access). The layers were mixed, and the ether (upper) layer was washed with 10% aq NaHCO_3_ and then H_2_O. The solvent was removed *in vacuo* (rotary evaporator). Absolute C_2_H_5_OH was added, and the drying operation was repeated twice.

The product (3-ketone) was dissolved in 10 ml of C_2_H_5_OH and then 0.05 mmol of NaB^3^H_4_ (25 mCi) was added. The solution was stirred at 23 °C for 2 h at 23 °C, and then 17 mg (0.45 mmol) of NaBH_4_ was added and stirring continued for 1.5 h. TLC analysis (silica, cyclohexane-ethyl acetate, 85-15, v-v) indicated that the reaction had gone to completion, yielding a ~9:1 ratio of dihydrolanosterol to epi-dihydrolanosterol (higher *R*_f_ spot). The solvent was removed under an N_2_ stream, and the residue was streaked on 2 mm × 20 mm × 20 mm preparative TLC (F_254_) plates, which were developed using a cyclohexane-ethyl acetate mixture (85-15, v-v) and visualized using 254 nm UV light. The lower *R*_f_ band was scraped and eluted through paper, followed by drying under an N_2_ stream. The overall yield (from dihydrolanosterol) was 98% (and 93% β-reduction), and subsequent analyzed TLC in the same solvent system indicated 99.8% radiopurity (of β-hydroxy dihydrolanosterol). The specific radioactivity was 7.55 mCi/mmol. The product was stored at -20 °C as an ethanolic solution (10 mM).

**Figure S20. Synthesis of [3-^3^H]-dihydrolanosterol.**

**[32-^2^H_3_]-Dihydrolanosterol** **(*d*_3_)** (Fig. S21) (19-21)

 **Figure S21. Synthesis of** **[32-^2^H_3_]-Dihydrolanosterol** **(*d*_3_)**

Compound **10** (Fig. S1, 1.2 g, 2.3 mmol) was added dropwise to 17 ml of *tert*-butanol containing 0.95 g of potassium *tert*-butoxide (at 23 °C). CD_3_I (1.46 ml, 3.33 g, 23.4 mmol) was added dropwise and the reaction proceeded for 16 h at 23 °C under Ar. About one-half of the solvent was removed *in vacuo* and diethyl ether was added. The ether phase was washed sequentially (2× each) with H_2_O, 5% aq HCl, and H_2_O and then dried with anhydrous MgSO_4_ and filtered through paper. The product showed a single major spot on TLC (silica, hexanes-ethyl acetate, 9:1, v-v, phosphomolybdic acid/heat). Crude yield 0.80 g (65%). HRMS (*m/z*) calcd for C_35_H_56_D_3_O_3_, [M+H]^+^, 530.4647; found, 530.4637 (Δ -1.9 ppm); calcd for C_35_H_54_D_3_O_2_, [M+H-H_2_O]^+^, 512.4541; found, 512.4532 (Δ -1.8 ppm).

The product was dissolved in 68 ml of diethylene glycol under Ar, and 32 ml of hydrazine hydrate (64-65%) was added dropwise, in a modified Wolff-Kishner reaction (20). After heating and stirring for 4 h at 150 °C, the reaction was cooled, and 2.7 g of KOH pellets was added. The temperature was raised to ~ 200 °C and ~ 20 ml of H_2_O was distilled off (using a distilling head placed before the condenser). Heating was continued at 220 °C, with stirring (under Ar) for 16 h.

The heat in the oil bath was turned off and 50 ml of H_2_O was added dropwise, with stirring. After 30 min, the reaction was cooled and dissolved in CH_2_Cl_2_. The organic layer was washed sequentially (3× each time) with H_2_O, 5% aq HCl, brine (saturated aq. NaCl), and H_2_O, dried with Na_2_SO_4_, and filtered through paper.

The product was dissolved in a minimum volume of CH_2_Cl_2_ and applied to a 2.0 cm × 30 cm silicic acid column. The column was eluted, in order, with hexanes and hexanes containing 3%, 8%, and 20% ethyl acetate (v/v). Aliquots of fractions were monitored with TLC (silica, hexanes-ethyl acetate, 4:1, v-v, phosphomolybdic acid/heat). Dihydrolanosterol eluted in the 20% ethyl acetate fraction. LC-HRMS analysis showed a major peak with *m/z* 414.4165 (C_30_H_48_D_3_, [M+H-H_2_O]^+^, Δ 3.3 ppm) plus a minor peak at *m/z* 397.3945 (C_29_H_49_). The latter corresponds to Compound **10** that had not reacted with CD_3_I but was reduced in the Wolff-Kishner reaction and lost the pivaloyl ester in the associated hydrolysis (i.e., 14-desmethyl dihydrolanosterol). This impurity (and also dihydroagnosterol) was removed by preparative HPLC (10 mm × 250 mm C_18_, 5 µm, 100% CH_3_CN). The deuterium content of the [32-^2^H_3_] dihydrolanosterol was estimated to be ≥ 98% as judged by HRMS, in line with the isotopic purity of the CD_3_I reagent used.

**epi-Dihydrolanosterol**

Dihydrolanosterol (0.80 g, 1.9 mmol), recrystallized as described above, was dissolved in 20 ml of CH_2_Cl_2_ and reacted with 0.80 g (2.0 mmol) of Dess-Martin periodinane for 1.5 h (67). The reaction was not complete, as judged by TLC (cyclohexane-ethyl acetate, 85:15, v-v). Stirring was continued (1 h) after the addition of another 0.80 g of periodinane (plus 10 ml CH_2_Cl_2_), after TLC showed the reaction was complete. Diethyl ether (100 ml) was added, and the reaction was decanted with 100 ml of saturated aq. NaHCO_3_ containing 2.2 g of Na_2_S_2_O_3_ (7-fold molar excess). After shaking, the (upper) ether layer was recovered and washed with saturated NaHCO_3_ and then H_2_O. The ether was removed *in vacuo* and the residue was dried and dissolved in 150 ml of C_2_H_5_OH. NaBH_4_ (150 mg, 4.5 mmol) was added, and the reaction was stirred for 2.5 h, after which TLC indicated that it had gone to completion (silica, cyclohexane-ethyl acetate: 85-15, v/v). The C_2_H_5_OH was removed *in vacuo*, and the residue was dissolved in a minimal amount of CH_2_Cl_2_ and applied to a 2.0 cm × 30 cm silicic acid column, which was washed with 100 ml hexanes and then 500 ml each of 4%, 6%, and 8% ethyl acetate in hexanes (v/v). Fractions of 20 ml were collected and analyzed by TLC (silica, cyclohexane-ethyl acetate, 85:15, v-v; visualization with phosphomolybdic acid spray and heating). The α-OH product (higher *R*_f_) (epi-dihydrolanosterol) eluted with 4% ethyl acetate and the β-OH product (lower *R*_f_) (dihydrolanosterol) eluted later with 4% and then with 6% ethyl acetate (in hexanes, v/v). The overall yields of epi-dihydrolanosterol (40.3 mg) and dihydrolanosterol (466 mg) were 5 and 58%, respectively.

**Agnosterol**

An impurity present in all commercial preparations of lanosterol and all dihydrolanosterol preparations was not removed by silica gel chromatography or preparative TLC but migrated earlier on a reversed-phase HPLC column and was identified as agnosterol (or dihydroagnosterol) by its *m/z* in mass spectrometry (2 a.m.u. less than lanosterol or dihydrolanostereol) and its characteristic UV spectrum (Figs. S22, S23). The ε_242_ value for this contaminant (18,600 M^-1^ cm^-1^) (28) allowed quantitation of this in dihydrolanosterol preparations and all derivatives (~ 2%), a value which was corroborated by LC-radioactivity measurements in the analysis of [3-^3^H]-dihydrolanosterol.

This impurity could be removed by preparative HPLC, but such purified materials did not yield rates of oxidation different than those with 2% dihydroagnosterol present.

**Figure S22. LC-UV profile of commercial lanosterol.** The sample was purchased from American Radiolabeled Chemicals. t_R_ 7.7 min, agnosterol; t_R_ 9.0 min, lanosterol; t_R_ 11.3 min, dihydroagnosterol; t_R_ 14.0 min, dihydrolanosterol.

**Figure S23. UV spectrum of agnosterol (from UPLC-diode array, Fig. S22).**

**Kinetic analysis**

**Txt files for analysis**

***k*_cat_, *K*_m_ determinations (for rates of FF-MAS formation)**

Dihydrolanosterol (L):

[L, µM "Rate, min-1" (replicates)

0.00 0.0 0.0 0.0

0.25 0.9 1.0 1.0

0.50 2.4 2.1 2.4

0.75 3.4 3.0 3.7

1.00 4.1 4.3 3.4

1.50 4.8 6.3 5.8

2.50 12.8 12.1 9.4

5.00 16.4 15.6 15.6

7.50 21.8 17.6 18.3

10.00 20.3 21.5 22.5

15.00 19.1 23.3 21.3

14-CH_2_OH dihydrolanosterol (14-OH L):

"[14-OH L],µM" "Rate, min-1” (replicates)

0.00 0.0 0.0 0.1

0.25 1.4 1.5 1.4

0.50 3.1 3.0 3.0

0.75 4.7 4.5 3.9

1.00 6.5 6.5 7.1

1.50 8.9 8.6 11.1

2.50 16.2 16.4 16.5

5.00 25.1 20.7 25.9

7.50 25.1 26.3 27.2

10.00 26.4 27.4 26.6

15.00 29.2 33.0 31.0

14-CHO dihydrolanosterol (14-CHO L):

[14-CHO L],µM "rate, min-1" (replicates)

0 0.013716 0.003597 0.000549

0.25 2.969398 3.527676 3.125914

0.5 7.815167 6.138808 6.65801

0.75 11.24817 9.099 10.0823

1 13.40152 13.67493 13.30145

1.5 18.85062 19.73997 19.25357

2.5 28.45919 30.66581 32.21464

5 52.7446 50.40213 54.72949

7.5 65.76442 62.40771 66.10811

10 70.12085 63.34467 67.78115

15 72.18041 76.96574 69.96132

***K*_d_ determinations** (L: dihydrolanosterol, L: dihydrolanosterol, epi-L: epi-dihydrolanosterol, 32-OH L: 14-CH_2_OH dihydrolanosterol; 14-CHO-L: 14-CHO dihydrolanosterol; FF-MAS, dihydro FF-MAS; all concentrations in µM)

**
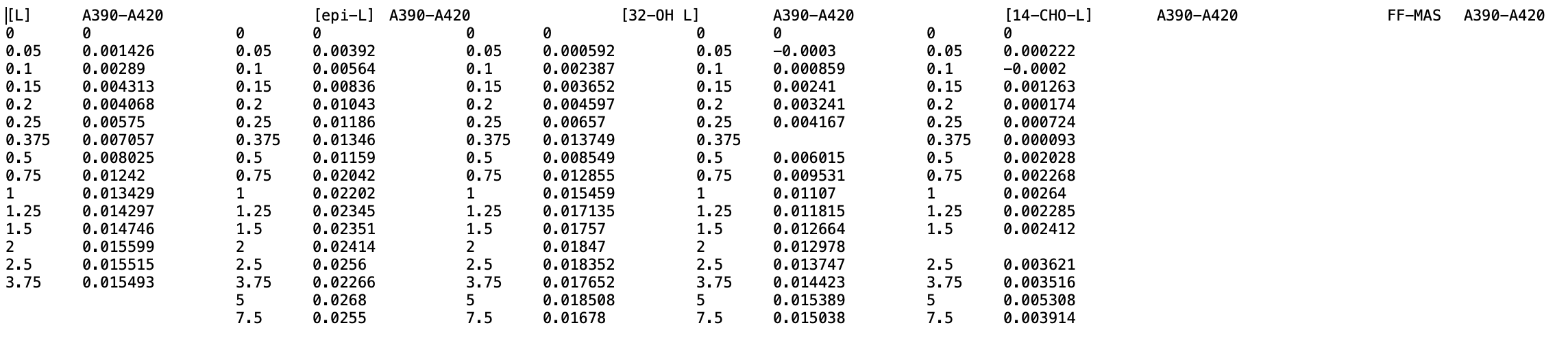
**

**Single turnover kinetics**

**Figure S24. Radio-HPLC trace (0.15 s time point).**

**Single turnover kinetics concentration txt files**

Time in s; S1: dihydrolanosterol, S2: 14-alcohol, S3: aldehyde, S4; dihydro FF-MAS (in µM)

Time S1 S2 S3 S4

0 7 0 0 0

0.05 4.7572 2.2428 0 0

0.1 2.9911 3.1584 0.8505 0

0.15 2.8308 2.7482 1.1396 0.2821

0.2 1.974 2.961 1.6401 0.4249

0.3 2.0671 2.3331 1.848 0.7518

0.4 1.9166 1.9978 1.8837 1.2019

0.6 1.5253 1.1711 1.7976 2.506

0.8 1.5841 0.9849 1.4126 3.0184

1 1.0563 0.4431 1.4476 4.0537

1.2 1.1711 0 1.3349 4.494

1.6 1.3601 0 0.8218 4.8181

2 1.0864 0 0.7105 5.2031

3 1.1137 0 0 5.8863

4 0.3647 0 0 6.6353

7 0 0 0 7

**A**

**
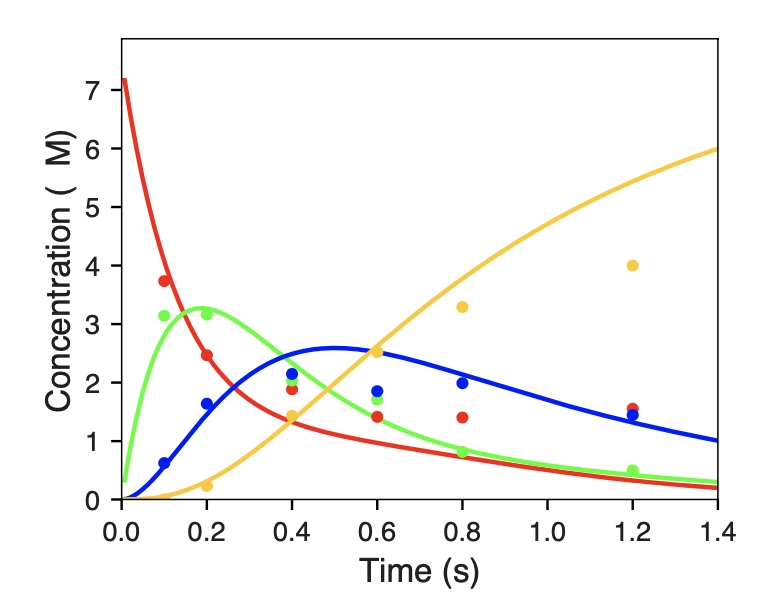
**

**B**

**
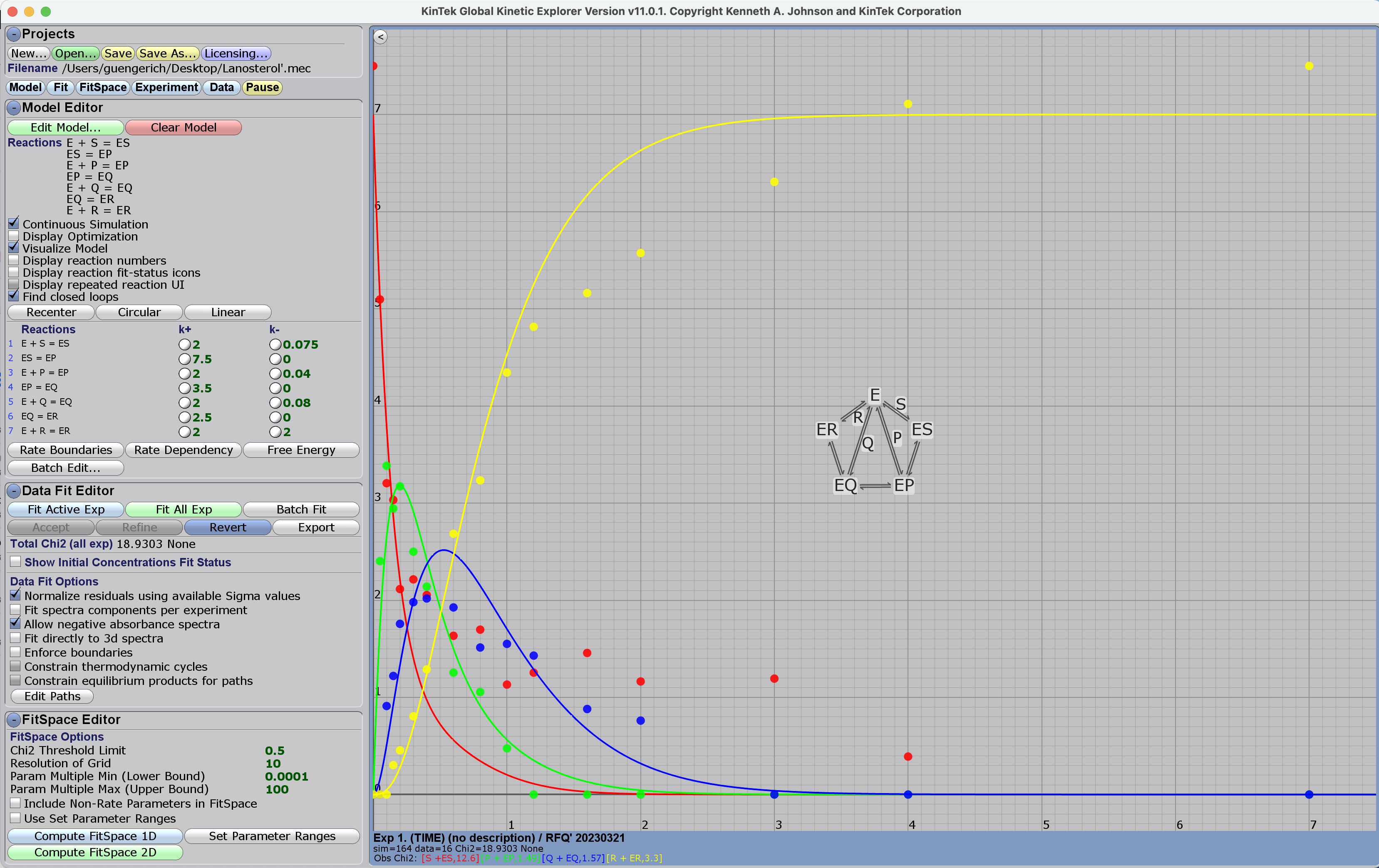
**

**C**

**
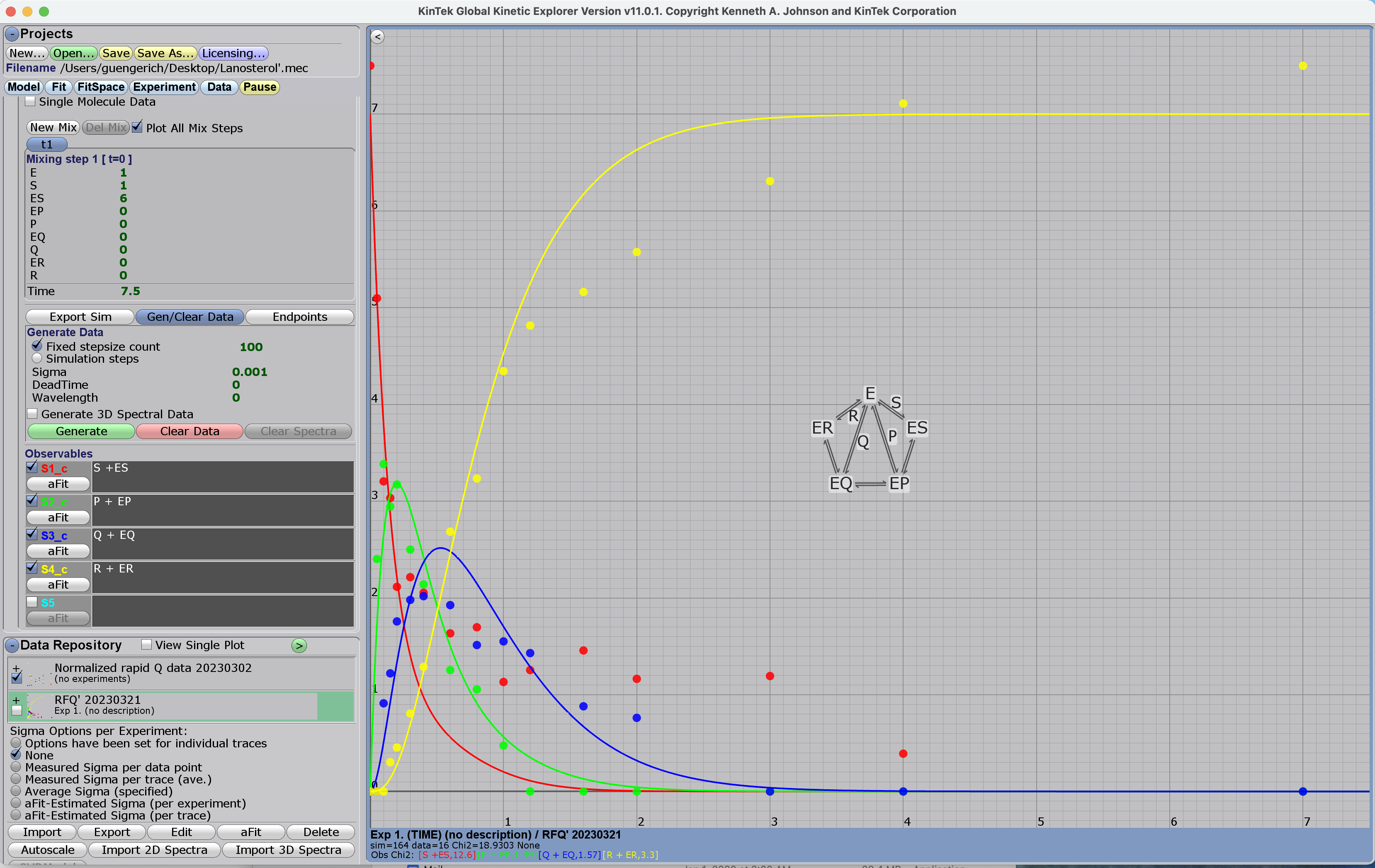
**

**Figure S25. Screen shots for KinTek Explorer fitting of rapid quench data**

*A*, preliminary run. Parts *B* and *C* show the various parameters on the left panels for the experiment presented in the text (the data points and the fitting (right panels) are identical. Time in s; S1: dihydrolanosterol (red), S2: 14-alcohol (green), S3: aldehyde (blue), S4; dihydro FF-MAS (yellow) (in µM).

**References** (21-23, 28 used in main text)

21. Takano, Y., and Morisaki, M. (1991) Efficient preparation of 32-oxygenated lanosterol derivatives. *Chem. Pharm. Bull.* **39**, 1647-1648

22. Morisaki, M., Igata, T., and Yamamoto, S. (2000) Synthesis of 15α-fluoro-24,25-dihydrolanosterol as a potential inhibitor and/or mechanistic probe for lanosterol 14α-demethylase. *Chem. Pharm. Bull.* **48**, 1474-1479

23. Araki, S., Eguchi, S., and Morisaki, M. (1990) Efficient entry to the steroidal 14α-methyl-8-ene system. *Chem. Pharm. Bull.* **38**, 1796-1797

28. Ruan, B., Watanabe, S., Eppig, J. J., Kwoh, C., Dzidic, N., Pang, J., Wilson, W. K., and Schroepfer, G. J., Jr. (1998) Sterols affecting meiosis: novel chemical syntheses and the biological activity and spectral properties of the synthetic sterols. *J. Lipid Res.* **39**, 2005-2020

65. Fieser, L. F., and Fieser, M. (1967) *Reagents for Organic Synthesis, Vol. 1.* Wiley, New York, pp 581-595

66. Corey, E. J., and Suggs, J. W. (1975) Pyridinium chlorochromate. An efficient reagent for oxidation of primary and secondary alcohols to carbonyl compounds. *Tetrahed. Lett.* **16**, 2647-2650

67. Dess, D. B., and Martin, J. C. (1983) Readily accessible 12-I-5 oxidant for the conversion of primary and secondary alcohols to aldehydes and ketones. *J. Org. Chem.* **48**, 4155-4156
